# Cortactin and fascin-1 regulate extracellular vesicle release by controlling endosomal trafficking or invadopodia formation and function

**DOI:** 10.1038/s41598-018-33868-z

**Published:** 2018-10-23

**Authors:** Els Beghein, Delphine Devriese, Evy Van Hoey, Jan Gettemans

**Affiliations:** Department of Biomolecular Medicine, Faculty of Medicine and Health Sciences, Campus Rommelaere, A. Baertsoenkaai 3, Ghent University, Ghent, Belgium

## Abstract

Cancer cell-derived extracellular vesicles (EVs) are increasingly being recognized as genuine invasive structures as they contribute to many aspects of invasion and metastasis. Unfortunately, the mechanisms underlying EV biogenesis or release are still poorly understood. Recent reports however indicate a role of the actin cytoskeleton in this process. In this study, we have exploited thoroughly characterized camelid nanobodies against actin binding proteins cortactin and fascin-1, a branched actin regulator and actin bundler, respectively, in order to assess their roles in EV biogenesis or release. Using this strategy, we demonstrate a role of the cortactin NTA and SH3 domains in EV release. Fascin-1 also regulates EV release, independently of its actin-bundling activity. We show a contribution of these protein domains in endosomal trafficking, a crucial step in EV biogenesis, and we confirm that EVs are preferentially released at invadopodia, the latter being actin-rich invasive cell protrusions in which cortactin and fascin-1 perform essential roles. Accordingly, EVs are enriched with invadopodial proteins such as the matrix metalloproteinase MT1-MMP and exert gelatinolytic activity. Based on our findings, we report that both cortactin and fascin-1 play key roles in EV release by regulating endosomal trafficking or invadopodia formation and function.

## Introduction

Extracellular vesicles (EVs) are nanometer-sized vesicles secreted by myriad of cells. EVs mediate local and systemic cell-cell communication through horizontal transfer of proteins, mRNAs and ncRNAs^1^. Living cells actively shed two distinct sets of EVs, i.e. microvesicles (100–1000 nm) and exosomes (50–200 nm). Microvesicles directly bud from the plasma membrane, whereas exosomes are formed intracellularly within multivesicular bodies (MVBs) as intraluminal vesicles (ILVs) and are released by fusion of these MVBs with the cell membrane^2^. Over the last decade, cancer-cell derived EVs gained more attention as they contribute to the processes of invasion and metastasis in many ways, including stimulating invasive growth, angiogenesis, chemoresistance, establishing the pre-metastatic niche and suppressing the immune response^3^. Consequently, EVs are being recognized as genuine invasive structures^4^.

Other mediators of the invasive cell phenotype are invadopodia^5^. These finger-like, actin-rich ventral cell protrusions are responsible for pericellular matrix degradation, which is mediated by focal delivery of matrix metalloproteinases (MT1-MMP, MMP-2 and MMP-9)^6^. Invadopodia thus endow cancer cells with the ability to breach through the extracellular matrix (ECM) and basement membrane, hence supporting invasive migration and metastasis. Invadopodia formation is a complex process and requires an intricate interplay between a distinct set of signalling proteins and actin binding proteins (ABPs), necessary for correct actin assembly and branching^7^.

Cortactin (CTTN) is a key branched actin regulator and scaffolding protein, linking signalling, membrane trafficking and other ABPs to dynamic actin networks^8^. CTTN regulates branched actin assembly via synchronised binding with the actin-nucleating ARP2/3 complex and filamentous actin (F-actin), at its N-terminal acidic (NTA) domain and 4^th^ repeat domain, respectively. The C-terminus of CTTN comprises a Src-homology (SH3) domain that serves as a docking place for a large number of proline-rich interacting proteins, including dynamin-2^9^, N-WASP^10^ and WIP^11^. The latter two proteins enhance ARP2/3-mediated actin polymerization^10,11^. Besides its role in invadopodia formation and maturation^12^, CTTN has well-established functions in endocytosis^13–19^ and endocytic trafficking^20–22^.

Fascin-1 (FSCN1) is an actin bundling protein. The protein specifically bundles parallel (unipolar) actin filaments, resulting in straight and compact bundles and providing mechanical stiffness^23^. Three actin binding sites were reported, which allow FSCN1 to bind and bundle at least two actin filaments^24,25^. FSCN1 was only recently detected in invadopodia where it acts as a stabilizer, enabling long invadopodium lifetime^12,24^.

We and others have used nanobodies (Nbs or VHHs; Variable domain of Heavy chain of Heavy chain antibodies) or single-domain antibodies as research tools to investigate protein function and to sort out molecular pathways^12,26–28^. The unique biochemical and biophysical properties of nanobodies, purportedly render them superior to antibodies or antibody-fragments. Moreover, nanobodies can be engineered in such a way that they display a desired function or set of functions, in order to expand their usefulness. Nanobodies can be used to study functions of structural (‘undruggable’) proteins and can be expressed in cells with the purpose of knocking out protein functions^28^. We previously generated stable MDA-MB-231 breast cancer cells with doxycycline (dox)-inducible expression of thoroughly characterized camelid nanobodies targeting CTTN SH3 or NTA domain (CTTN SH3 Nb2 or CTTN NTA Nb2^12,26^, respectively) or FSCN1 (FSCN1 Nb5^12,29^). FSCN1 Nb5 was additionally coupled to an N-terminal mitochondrial outer membrane (MOM)-tag (MOM-FSCN1 Nb5) which limits free diffusion of endogenous FSCN1 at the outer mitochondrial membrane and thus displaces the protein from where it is needed^12,28^. All nanobodies have well-established effects on actin polymerization and bundling, and accordingly on invadopodia organization, formation, stabilization and maturation^12,26^.

EV biogenesis or release is very poorly understood, although mechanisms that regulate the actin cytoskeleton are somehow involved in this process^30^. Moreover, recent literature reports on a synergistic interaction between invadopodia biogenesis and EV release^31^, and CTTN being involved in MVB docking^32^. These observations prompted us to elucidate CTTN’s and FSCN1’s contribution to EV release, using the abovementioned highly characterized camelid nanobodies.

In this study, we prove a role of the CTTN NTA domain and demonstrate for the first time a role of the CTTN SH3 domain in EV release. The actin bundler FSCN1 also regulates EV release, albeit independently of its actin-bundling activity. We assess the contribution of these protein domains in endosomal trafficking, a key step in EV biogenesis, and we confirm the close interconnection between invadopodia and EV release. In fact, when inducing an invadopodia pattern change, MVB organization changes equally. Moreover, MDA-MB-231 EVs are enriched with invadopodial proteins, such as the invadopodia marker MT1-MMP, and possess gelatinolytic activity. Finally, our data emphasize the importance of discerning genuine EV-regulating from invadopodia-regulating proteins in future research.

## Results

### MDA-MB-231 OptiPrep density gradient (ODG)-purified EVs are enriched with EV-markers and lack contaminating proteins

**Figure 1:**
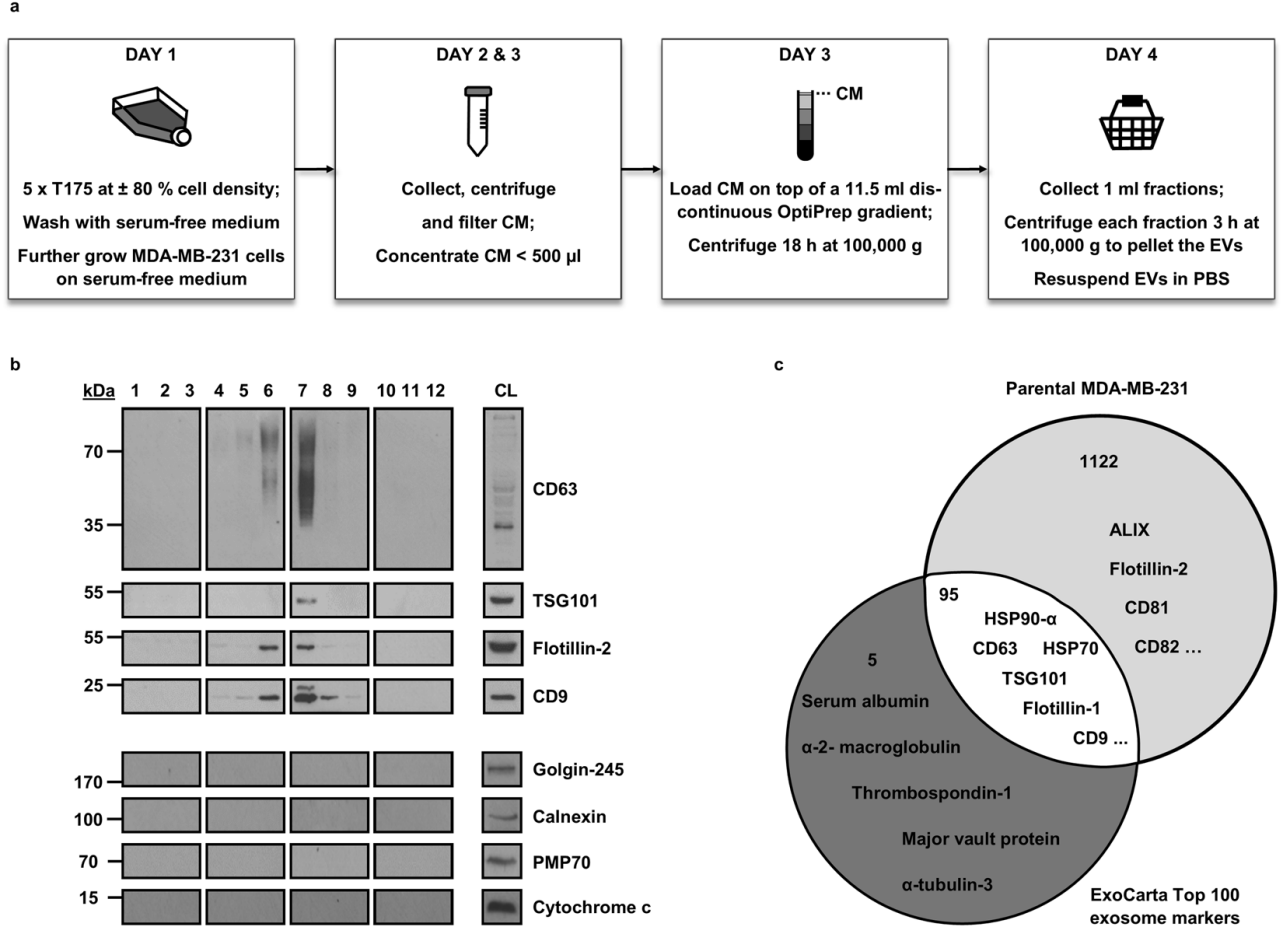
EV isolation protocol flow chart and validation of the technique. (**a**) EV isolation flow chart. MDA-MB-231 are grown until a density of ±80% is reached. Subsequently, cells are washed and the culture medium is replaced by its serum-free counterpart. On days 2 and 3, the conditioned medium (CM) is harvested, centrifuged and filtered to remove dead cells, large vesicles and debris. Next, the CM is concentrated to <500 µl using ultrafiltration and loaded on top of a 11.5 ml discontinuous OptiPrep gradient. Following 18 h ultracentrifugation, 1 ml fractions are harvested and centrifuged separately to pellet the EVs. Finally, EVs are resuspended in PBS and if desired, stored at −80 °C. (**b**) Validation of the isolation protocol by Western blot. EVs in the CM are separated according to their density after ODG centrifugation and are harvested in 12 discrete fractions. A representative Western blot of these fractions confirms the presence of EV-markers CD63, TSG101, flotillin-2 and CD9 mainly in fractions 6 and 7 (EV+ fractions). All fractions were clear from contaminating cell organelles as indicated by the absence of markers of the Golgi apparatus (golgin-245), endoplasmic reticulum (calnexin), peroxisomes (PMP70) and mitochondria (cytochrome c). CL = crude lysate. For reasons of clarity and conciseness, blots were cropped to the bands of interest. Samples derive from the same experiment and gels/blots were processed in parallel. Full-length blots are presented in Supplementary Figure S10. (**c**) Validation of the isolation protocol by shotgun MS. Venn diagram of proteins detected by MS in EV+ fractions 6 and 7 versus exosome markers listed in the ExoCarta Top 100 (published on the ExoCarta database http://exocarta.org/). 1,217 different proteins were detected in the EV+ fractions (light grey plus white section), of which 95 proteins (white intersection) also occur in the ExoCarta Top 100 list. Serum albumin and α-2-macroglobulin (dark grey section) were absent in MDA-MB-231 EV+ fractions but are most likely contaminating proteins. Additional to the proteins detected by Western blot, EV-markers flotillin-1, HSP90-α, HSP70, CD82, CD81 and ALIX were identified in the EV+ fractions (light grey and white section).

EVs were isolated from pre-purified conditioned medium (CM) derived from parental MDA-MB-231 breast cancer cells by means of OptiPrep density gradient (ODG) centrifugation. The protocol is explained in detail in the Methods Section and is succinctly outlined in Fig. 1a. In a nutshell, ODG centrifugation separates MDA-MB-231 secretome according to density, ultimately resulting in 12 different density fractions. Western blot analysis shows enrichment of EV-marker proteins CD63, TSG101, flotillin-2 and CD9 in fraction 6 and 7 (Fig. 1b), which are further referred to as EV-positive (EV+) fractions throughout this manuscript. These fractions cover densities ρ 1.088 g/ml and 1.122 g/ml, analogous to previously reported densities using ODG^33–35^. Nanoparticle tracking analysis confirmed presence of a high concentration (average ± 4 × 10^3^ particles/cell) of ±86–151 nm-sized particles (see Supplementary Dataset File 1), as what is expected when using nanoparticle tracking analysis post-ODG centrifugation^32,34,35^. All fractions were clear from contaminating cell organelle markers golgin-245 (Golgi apparatus), calnexin (endoplasmic reticulum), PMP70 (peroxisomes) and cytochrome c (mitochondria), thus excluding cell lysis and apoptosis. Shotgun mass spectrometry (MS) of EV+ fractions resulted in 1,217 unique proteins (see Supplementary Dataset File 2) and confirmed presence or absence of the abovementioned markers and contaminants, respectively (Fig. 1c and Supplementary Dataset File 2). When compared to the ExoCarta Top 100 list of exosome markers, 95 proteins out of 100 were retrieved in EV+ fractions, including HSP90-α, HSP70 and flotillin-1 (Fig. 1c). Of note, these fractions lack the ExoCarta-listed exosome markers serum albumin and α-2-macroglobulin, which are most likely contaminating serum proteins^34,36^. Organelle contaminants such as prohibitin (mitochondria), GM130 (Golgi apparatus) and calreticulin (endoplasmic reticulum)^34^ were not detected (See Supplementary Dataset File 2). Also worth mentioning is the presence of other EV-markers (ALIX, CD81 and CD82), several integrin subunits, actin, ABPs (a.o. FSCN1, vinculin, α-actinin-1 and 4, cofilin-1, moesin, ezrin, radixin and several subunits of the ARP2/3 complex), growth factor receptors (a.o. EGFR and TGFR), small GTPases (a.o. CDC42, RhoA, RhoC) and a number of α and β-tubulin subtypes (see Supplementary Dataset File 2). In conclusion, ODG-purified EV preparations derived from MDA-MB-231 are highly enriched with typical EV-markers and completely clear from contaminating cell organelles.

### Nanobodies affecting CTTN function or FSCN1 localization induce a decrease in EV release but have different effects on mean EV size

**Figure 2:**
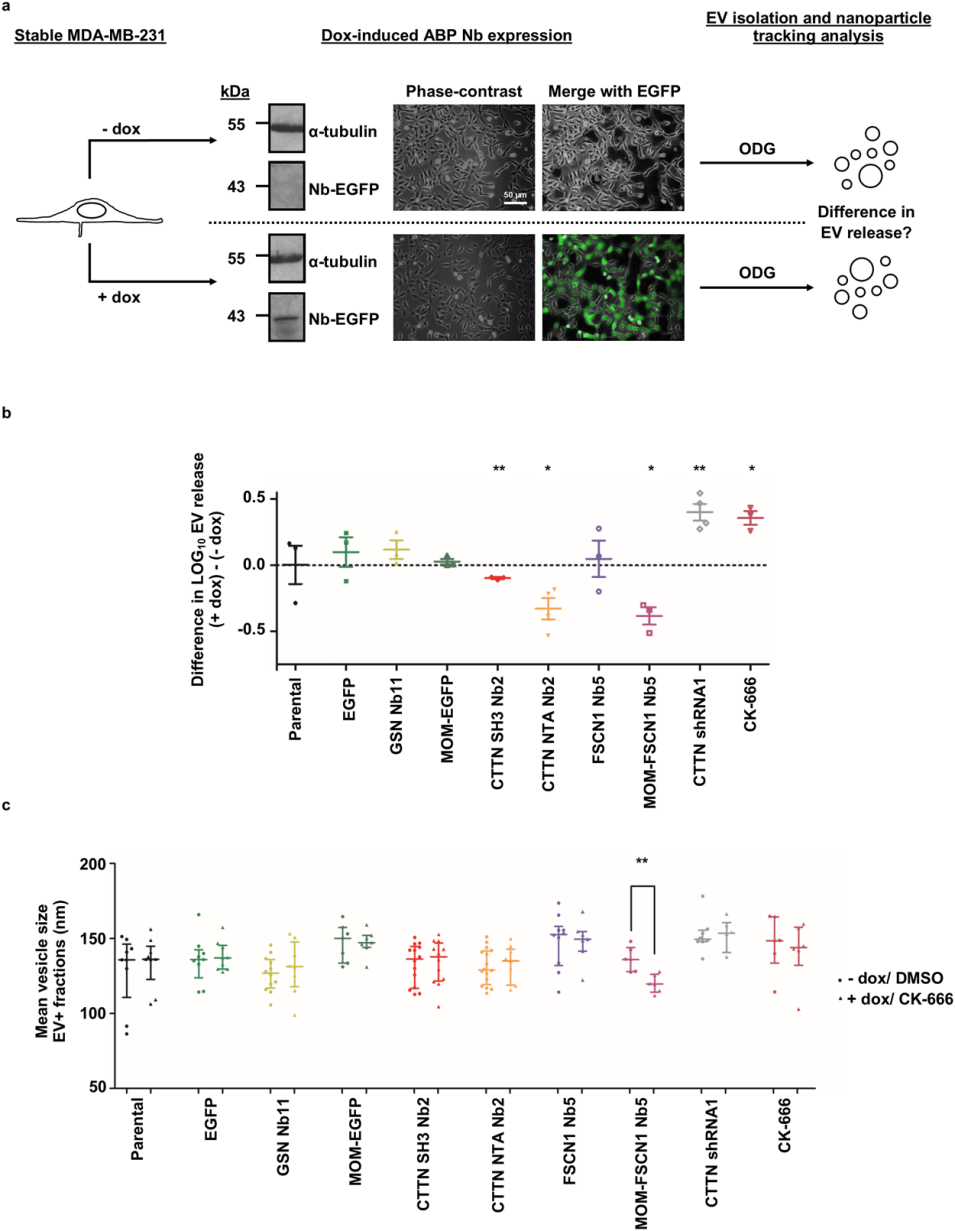
Nanobodies interfering with CTTN function or FSCN1 localization induce a decrease in EV release but have dissimilar effects on mean EV size. (**a**) EVs of MDA-MB-231 with dox-inducible EGFP-tagged nanobody (Nb) expression (24 h, 500 ng/ml dox) were isolated by means of ODG centrifugation and were subsequently quantified by nanoparticle tracking analysis. Scheme illustrates induction of ABP nanobody expression and pursued flow chart. Inducible nanobody expression is illustrated using GSN Nb11 MDA-MB-231. α-tubulin was used as a loading control. For reasons of clarity and conciseness, blots were cropped to the bands of interest. Full-length blots are presented in Supplementary Figure S10. (**b**) Graphs represent mean difference in LOG EV release (LOG_10_ particles/cell) between nanobody expressing (+dox) and non-expressing (−dox) cells with SEM (n $\ge$ 3). P_0.05_-value was determined by a ratio paired t test. Neither dox addition as such (p = 0.9914), nor EGFP (p = 0.4701) or MOM-EGFP (p = 0.3261) expression significantly alter EV release. Expression of a nanobody against the actin severing protein gelsolin (GSN Nb11) does not significantly affect EV release (p = 0.2363). EV release is significantly reduced by CTTN SH3 Nb2 and CTTN NTA Nb2 expression (p = 0.0062 and p = 0.0397, respectively). FSCN1 Nb5 does not alter EV release (p = 0.7624). Conversely, delocalization of FSCN1 towards the mitochondria (MOM-FSCN1 Nb5) significantly reduces EV release (p = 0.0276). On the other hand, CTTN knockdown by means of RNAi (CTTN shRNA1) or inhibition of the ARP2/3 complex by CK-666 (100 µM, 24 h) significantly induces EV release (p = 0.0204 and p = 0.0080, respectively). *P ≤ 0.05 and **P ≤ 0.01. (**c**) Quantification of mean vesicle size by nanoparticle tracking analysis. Dots or triangles represent mean vesicle size of single EV+ fraction measurements (n ≥ 6 each condition) of non-expressing (−dox)/DMSO-treated versus expressing (+dox)/CK-666-treated MDA-MB-231 cells, respectively. Graphs represent median vesicle size with interquartile range. P_0.05_-value was determined by a Mann-Whitney rank sum test. Only MOM-FSCN1 Nb5 expression significantly reduces mean EV size (p = 0.0043). **P ≤ 0.01.

We next set out to investigate the effects of ABP nanobody expression on EV release in MDA-MB-231. All ABP nanobodies have well-established effects on actin assembly; CTTN SH3 Nb2 hinders WIP binding^12^, CTTN NTA Nb2 interferes with ARP2/3-mediated actin polymerization^26^ and FSCN1 Nb5 impedes F-actin bundling^12^. Nanobody expression was induced by adding dox and induced cells were grown in parallel with non-induced matching pairs. EVs were isolated by means of ODG centrifugation and EV+ fractions were analysed using nanoparticle tracking analysis (Fig. 2a and see Supplementary Dataset File 1). Addition of dox as such has no effect on EV release, neither does expression of EGFP, MOM-EGFP or GSN Nb11 (a nanobody binding the actin severing protein gelsolin or GSN^37^) (Fig. 2b). Although FSCN1 Nb5 expression does not alter EV release, delocalization of endogenous FSCN1 towards the mitochondrial outer membrane (MOM-FSCN1 Nb5) significantly reduces EV release (p = 0.0276). Both CTTN SH3 Nb2 and CTTN NTA Nb2 significantly reduce EV release (p = 0.0062 and p = 0.0397, respectively). Conversely, a CTTN shRNA expressing cell line (CTTN shRNA1) that successfully down-regulates CTTN expression (±80% reduction, see Supplementary Figure S1), induces an increase in EV release (p = 0.0080) (Fig. 2b and see Supplementary Figure S2). Considering CTTN’s well-established function in endocytosis^13–19^, endocytic trafficking^20–22^, MVB docking and consequently, EV release^32^, this increase in EV release was unexpected. Next, we treated parental MDA-MB-231 with ARP2/3 complex-inhibitor CK-666. The ARP2/3 complex nucleates the formation of actin branches^38^ and its involvement in endocytosis^14,39–41^, endocytic dynamics^16,21,22^ and even EV release^32^ has been reported. To our surprise, CK-666 treatment significantly induces EV release (p = 0.0204) (Fig. 2b and see Supplementary Figure S2). Strikingly, both CK-666 treatment and CTTN shRNA1 expression significantly reduce cell numbers compared to their corresponding controls, but do not affect cell viability (see Supplementary Figure S3). Nanobody expression does not significantly alter cell counts and viability, nor does expression of EGFP, MOM-EGFP or addition of dox as such (see Supplementary Figure S3). Apart from some outliers, mean EV size of all cell lines typically ranges from ±90 to 160 nm (Fig. 2c and see Supplementary Dataset File 1). Only MOM-FSCN1 Nb5 expression significantly reduces EV size (p = 0.0043) compared to its non-induced counterpart. To determine whether nanobody expression could interfere with EV protein cargo composition, EV+ fractions were analysed by means of shotgun MS (see Supplementary Figure S4 and Supplementary Dataset File 3). Expression of CTTN NTA Nb2 and FSCN1 Nb5 does not interfere with cargo sorting. However, MS identified CD99 antigen-like protein 2 (CD99L2) in EVs purified from expressing CTTN SH3 Nb2 cells, but not in EVs purified from their non-expressing matching pairs. Moreover, 60S ribosomal protein L18 (RPL18) was no longer detected in EVs when MOM-FSCN1 Nb5 was expressed. Collectively, these results indicate that CTTN and FSCN1 positively regulate EV release in MDA-MB-231. Both SH3 and NTA domain of CTTN are involved in EV release, whereas FSCN1 regulates EV release and size, but independently of its actin-bundling activity. FSCN1, again independently of its actin-bundling function, and the CTTN SH3 domain may be implicated in EV protein sorting. We decided to exclude the CTTN shRNA 1 MDA-MB-231 cell line and CK-666 treatment from subsequent experiments, considering the questionable results.

### Nanobody-induced reduction in EV release seems related to CTTN or FSCN1 roles in endosomal maturation and/or invadopodia formation or maturation

**Figure 3:**
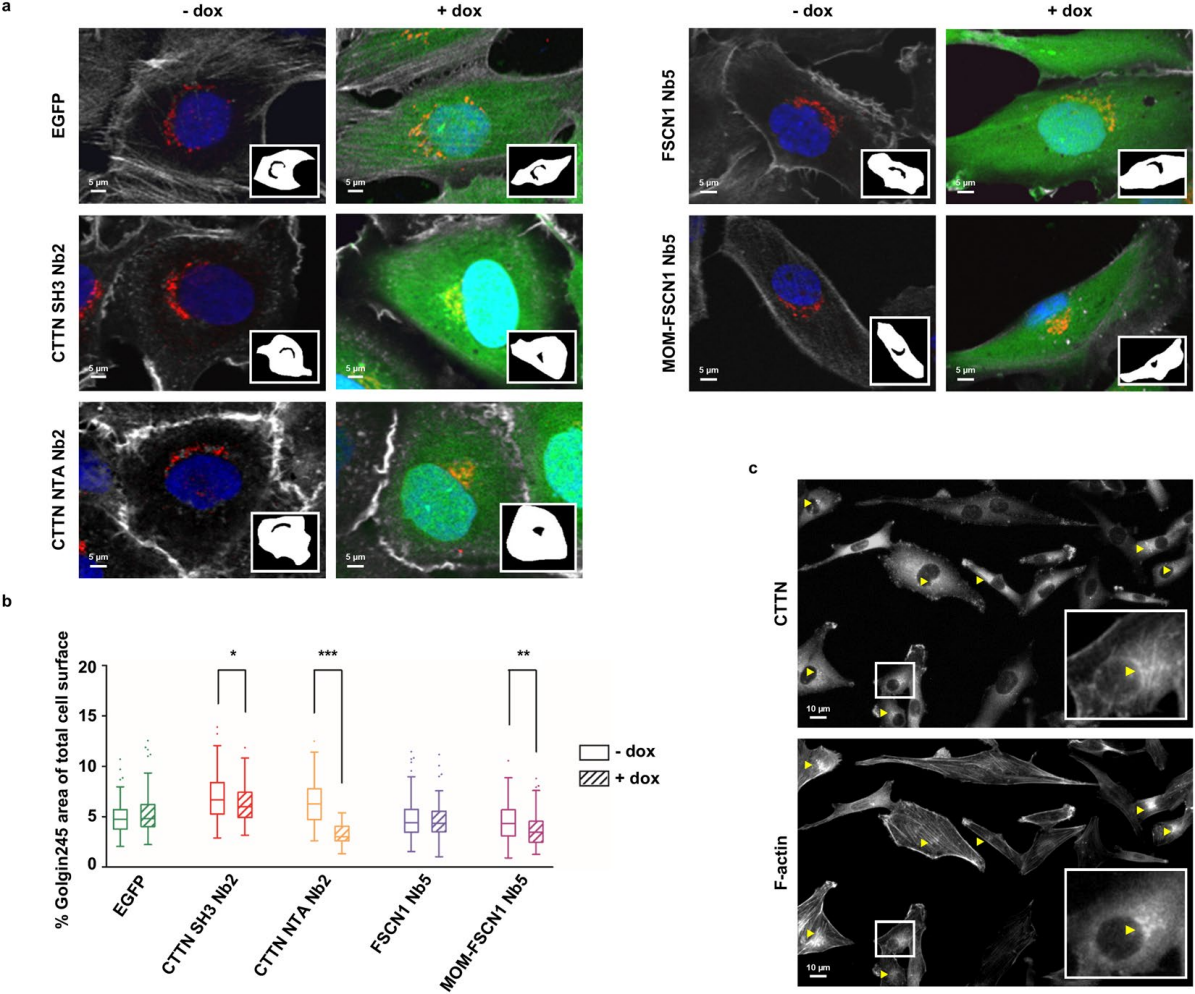
Nanobody-induced reduction in EV release could be ascribed to CTTN or FSCN1 roles in endosomal maturation and/or invadopodia formation or maturation. (**a**) Representative confocal images of MDA-MB-231 breast cancer cells stably expressing ABP nanobodies (+dox, 24 h induction with 500 ng/ml dox) and without nanobody expression (−dox). Unlike EGFP and FSCN1 Nb5 expression, CTTN SH3 Nb2, CTTN NTA Nb2 and MOM-FSCN1 Nb5 expression induce a compaction of the Golgi apparatus (red). Extracted Golgi areas are shown in the insets. Nuclei (blue) were visualized by means of DAPI, Golgi apparatus (red) with anti-golgin-245 antibody and cell cortex (grey) by means of phalloidin staining. Nanobodies (green) are intrinsically fluorescent via their EGFP-tag. (**b**) Graphs represent quantification of Golgi spread area by means of golgin-245 staining in nanobody-expressing versus non-expressing MDA-MB-231 cells. Tukey boxplots represent quantification of the ratio of total golgin-245 area to total cell area. P_0.05_-value was determined by a Mann-Whitney rank sum test and n ≥100 cells were quantified for each boxplot. A compacted Golgi morphology can be observed in cells expressing (+dox) CTTN SH3 Nb2 (p = 0.0370), CTTN NTA Nb2 (p < 0.0001) and MOM-FSCN1 Nb5 (p = 0.0023), compared to their non-expressing (−dox) corresponding cell lines. Expression of EGFP as such has no effect on Golgi morphology (p = 0.2559), nor does expression of FSCN1 Nb5 (p = 0.5183). *P ≤ 0.05, **p ≤ 0.01 and ***p ≤ 0.001. (**c**) Representative epifluorescence images of parental MDA-MB-231 cells seeded onto a polystyrene plate (48 h on serum-free medium). Invadopodia are visualized by immunofluorescence of CTTN and F-actin (Alexa fluor 594 phalloidin staining). Enrichment of both markers can be observed at the perinuclear region, which is typical of invadopodia. Boxed areas are enlarged in the bottom right insets and arrowheads Δ indicate areas of CTTN and phalloidin enrichment and colocalization.

Endosomal maturation is an essential step in EV biogenesis^2^ and interfering with this process can explain a reduction in EV release. CTTN controls late endosomal/lysosomal (LE/lys) maturation and consequent retrograde transport to the Golgi apparatus. Knock-down of CTTN leads to a striking compaction of the Golgi apparatus by light microscopy, presumably caused by disturbed lipid trafficking secondary to the maturation defects^22^. We set out to investigate whether our CTTN and FSCN1 nanobodies induce a similar Golgi compaction and thus whether they interfere with LE/lys maturation and trafficking. When compared to a EGFP-control, CTTN SH3 Nb2 moderately induces Golgi-compaction (p = 0.0370) (Fig. 3a,b). However, expression of CTTN NTA Nb2 dramatically alters Golgi morphology (p < 0.0001). MOM-FSCN1 Nb5 significantly reduces Golgi area (p = 0.0023), while this is not the case for FSCN1 Nb5. Of note, EGFP or ABP nanobody expression does not alter cell area (see Supplementary Figure S5). Secondly, a reduction in EV release could be caused by impeding CTTN and FSCN1 roles in invadopodia formation and function, since there is a synergistic interaction between invadopodia biogenesis and EV secretion^31^. CTTN and FSCN1 are essential proteins for invadopodia biogenesis^7^ and our ABP nanobodies induce well-established defects in invadopodia initiation, stabilization and maturation^12,26,29^. Immunostaining of parental MDA-MB-231 on polystyrene plates, grown in identical conditions as for EV isolation (Fig. 2), shows perinuclear enrichment of invadopodial proteins CTTN and F-actin (Fig. 3c), suggesting genuine invadopodia formation.

To conclude, these data confirm the involvement of CTTN in endosomal maturation, with both the SH3 and NTA domain playing a role. Moreover, we show a role of FSCN1 in endosomal maturation, independently of its actin-bundling activity. Since MDA-MB-231 cells form invadopodia during the EV isolation process, nanobody-effects on EV release can also be in part explained by their effects on invadopodia biogenesis, which is indirectly coupled to EV release.

### MVBs and invadopodia are closely interconnected

**Figure 4:**
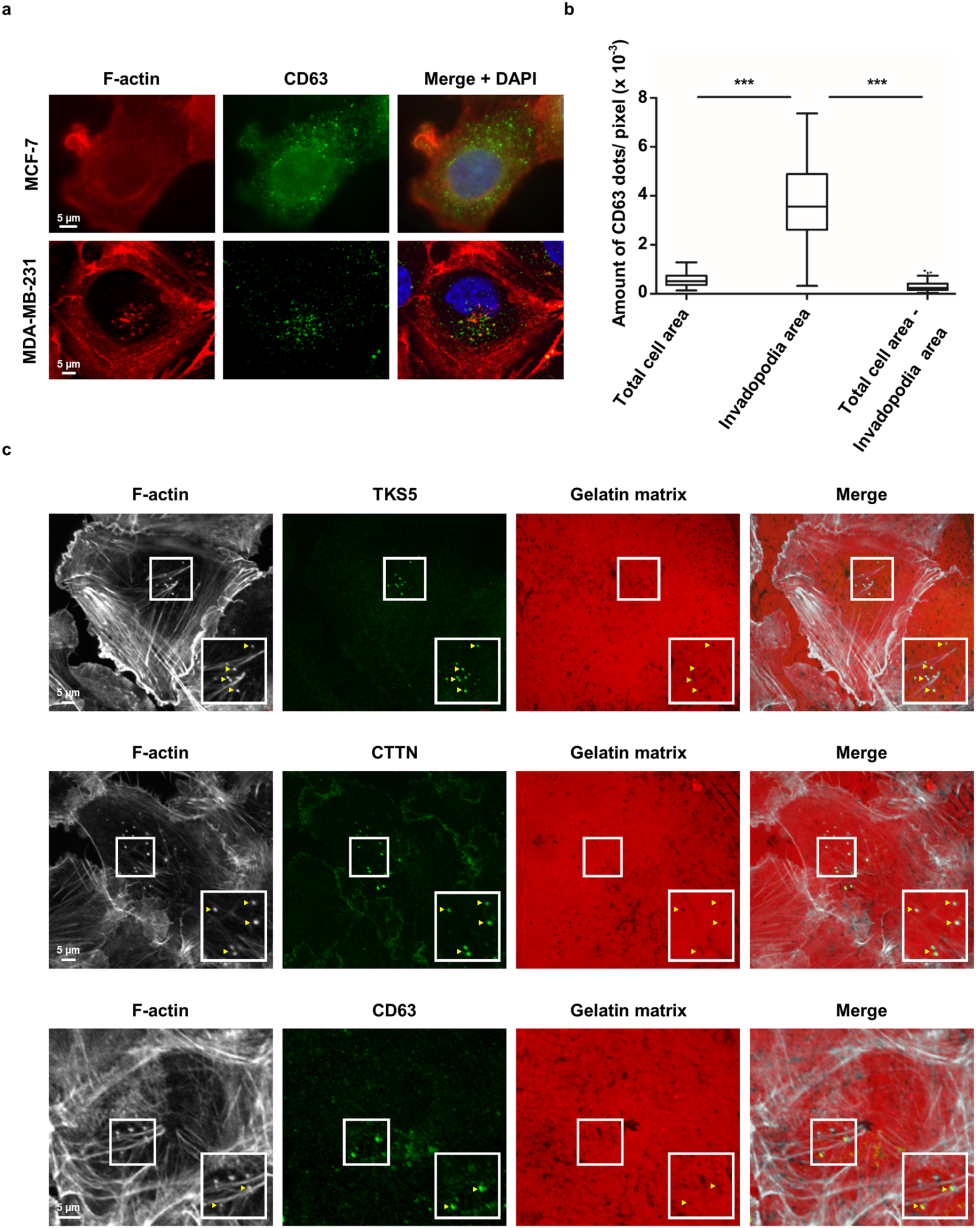
CD63+ vesicles typically localize near invadopodia in MDA-MB-231. (**a**) Representative epifluorescence images of F-actin (Alexa fluor 594 phalloidin staining) and CD63 in parental MCF-7 (top panels) and MDA-MB-231 (bottom panels) breast cancer cells seeded onto a gelatin matrix. MCF-7 cells do not form invadopodia (phalloidin, left top panel) and have a diffuse CD63-pattern (middle top panel). Conversely, MDA-MB-231 do form invadopodia which can be observed as perinuclear phalloidin dots (left bottom panel). The CD63 signal organizes in a very discrete region (middle bottom panel) and this region colocalizes with the perinuclear invadopodia region (right bottom panel). (**b**) Quantification of the CD63 dot density at the total cell area (left), invadopodia region (middle) and total cell area without the invadopodia region (right) in parental MDA-MB-231. Tukey boxplots represent quantification of the amount of CD63 dots per pixel. P_0.05_-value was determined by a Mann-Whitney rank sum test and n ≥100 cells were quantified for each boxplot. CD63 dot density is significantly higher at the invadopodia region compared to the total cell area (p < 0.0001), or compared to the cell area minus the invadopodia region (p < 0.0001), as illustrated in (**a**) (bottom panels). ***P ≤ 0.001. (**c**) Representative confocal images of parental MDA-MB-231 cells seeded onto a fluorescent gelatin matrix. Invadopodia are visualized by immunofluorescence of TKS5, CTTN and F-actin (Acti-Stain 670 phalloidin staining). Perinuclear F-actin dots colocalize with TKS5 (top panels) and CTTN (middle panels) respectively, and with areas of matrix degradation, confirming the presence of invasive invadopodia. CD63 on the other hand, occasionally colocalizes with F-actin and with areas of matrix degradation (invadopodia), but both signals are always near each other (bottom panels). Boxed areas are enlarged in the bottom right insets and arrowheads Δ indicate areas of colocalization.

It was previously shown that MVBs localize near and dynamically interact with invadopodia^31^. Figure 4a confirms MDA-MB-231 forming invadopodia on gelatin matrix, which can be observed as F-actin-rich perinuclear dots (Fig. 4a, left bottom panel). MVBs (CD63+ vesicles) organize in a well-delineated region (middle bottom panel) and this area colocalizes with the perinuclear invadopodia region (right bottom panel). This is not the case for MCF-7 breast cancer cells; these cells do not form invadopodia (Fig. 4a, left top panel) and the MVBs are diffusively dispersed throughout the cell (middle top panel). Quantification of the CD63 dot density in MDA-MB-231 confirms a much higher MVB density at the invadopodia area versus total cell area, or versus total cell minus invadopodia area (Fig. 4b). Colocalization of the perinuclear F-actin (phalloidin) dots with invadopodium markers TKS5, CTTN and dark areas of fluorescent gelatin matrix degradation, corroborates the formation of genuine invasive invadopodia (Fig. 4c, top and middle panels). CD63+ MVBs occasionally colocalize with invadopodia, but both are always near each other (Fig. 4c, bottom panels).

**Figure 5:**
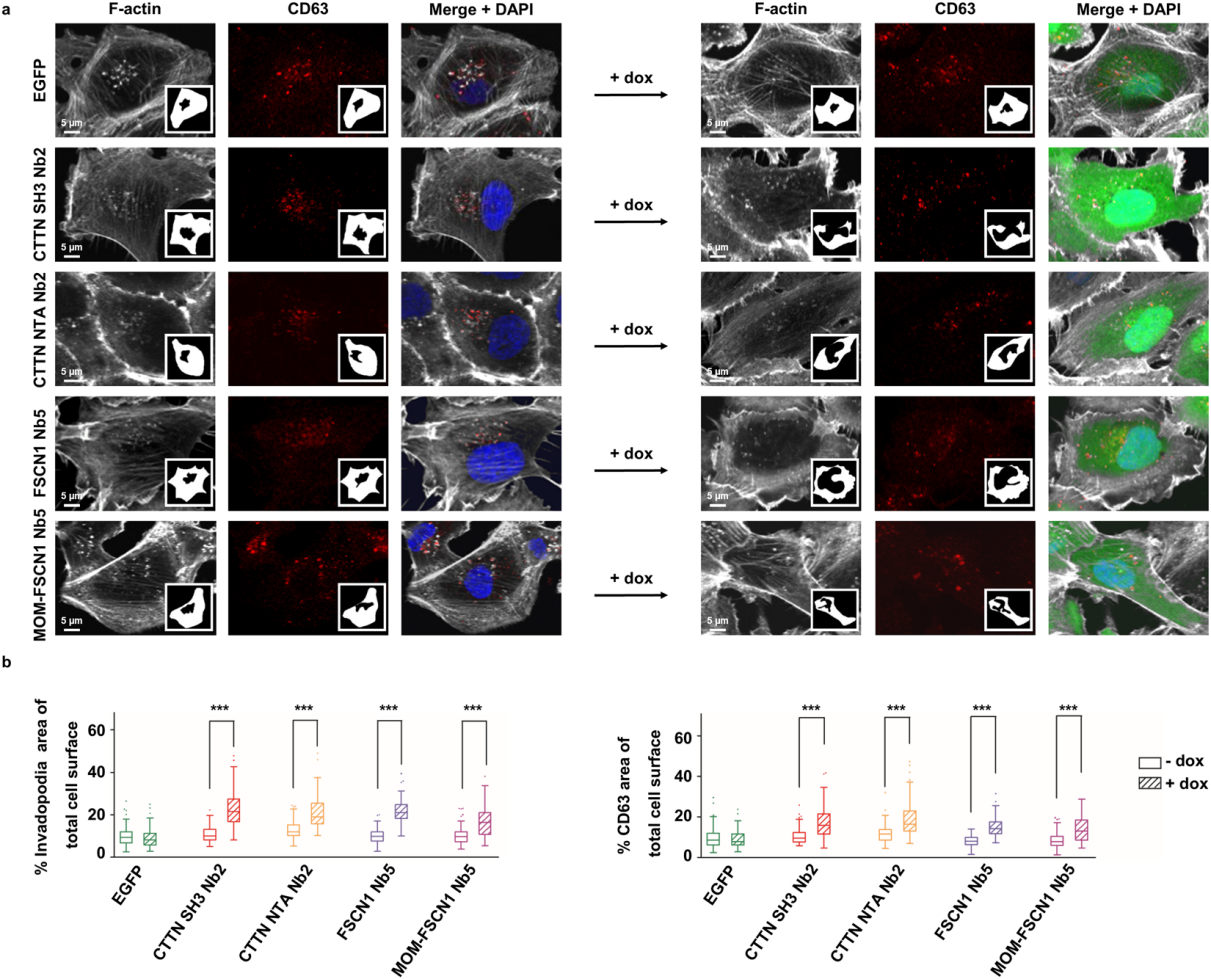
Nanobodies interfering with CTTN and FSCN1 function or FSCN1 localization affect invadopodia and CD63+ vesicle pattern determination. (**a**) Representative confocal images of MDA-MB-231 breast cancer cells stably expressing ABP nanobodies (right panels, 24 h induction with 500 ng/ml dox) and without nanobody expression (left panels). All nanobodies induce an increase in perinuclear F-actin (Acti-Stain 670 phalloidin) spread area (first panels on the left versus first panels on the right). Nanobody expression has a similar effect on the CD63 pattern (middle panels on the left versus middle panels on the right). EGFP expression as such neither alters the F-actin pattern, nor the CD63 pattern (top panels left versus top panels right). Extracted F-actin/invadopodia or CD63 areas are shown in the insets. Nuclei (blue) were visualized by means of DAPI and nanobodies (green) are intrinsically fluorescent via their EGFP-tag. (**b**) Quantification of invadopodia (left) and CD63 (right) spread area by means of Acti-Stain 670 phalloidin and CD63 staining, respectively, in nanobody-expressing (+dox, 24 h induction with 500 ng/ml dox) versus non-expressing (−dox) MDA-MB-231 cells. Tukey boxplots represent quantification of the ratio of total perinuclear phalloidin area to total cell area (left) or the ratio of total CD63 area to total cell area (right). P_0.05_-value was determined by a Mann-Whitney rank sum test and n ≥100 cells were quantified for each boxplot. The boxplot data corroborate the microscopy data in (**a**). P-values phalloidin area: 0.1643 (EGFP), <0.0001 (CTTN SH3 Nb2, CTTN NTA Nb2, FSCN1 Nb5 and MOM-FSCN1 Nb5). P-values CD63 area: 0.5489 (EGFP), <0.0001 (CTTN SH3 Nb2, CTTN NTA Nb2, FSCN1 Nb5 and MOM-FSCN1 Nb5). ***P ≤ 0.001.

ABP nanobody expression affects normal invadopodia pattern formation, giving rise to loosely packed invadopodia that occupy more space than sheer perinuclear area^12^. We wondered if the MVB pattern would follow this change in invadopodia spread pattern. ABP nanobody expression indeed induces a significant increase in invadopodia (F-actin) spread area, compared to an EGFP control (Fig. 5a, panels on the left versus panels on the right). CD63 + MVB spread area also expands when ABP nanobodies are expressed (Fig. 5a). Quantification of invadopodia and MVB spread area corroborates an enlargement of both invadopodia and MVB area (Fig. 5b). Moreover, in ABP nanobody-expressing cells, MVBs still localize near invadopodia (see Supplementary Figure S6). Taken together, these results confirm a close spatial interconnection between MVBs and invadopodia in MDA-MB-231.

### MDA-MB-231 EVs exhibit gelatinolytic activity presumably mediated by matrix metalloproteinase MT1-MMP

**Figure 6:**
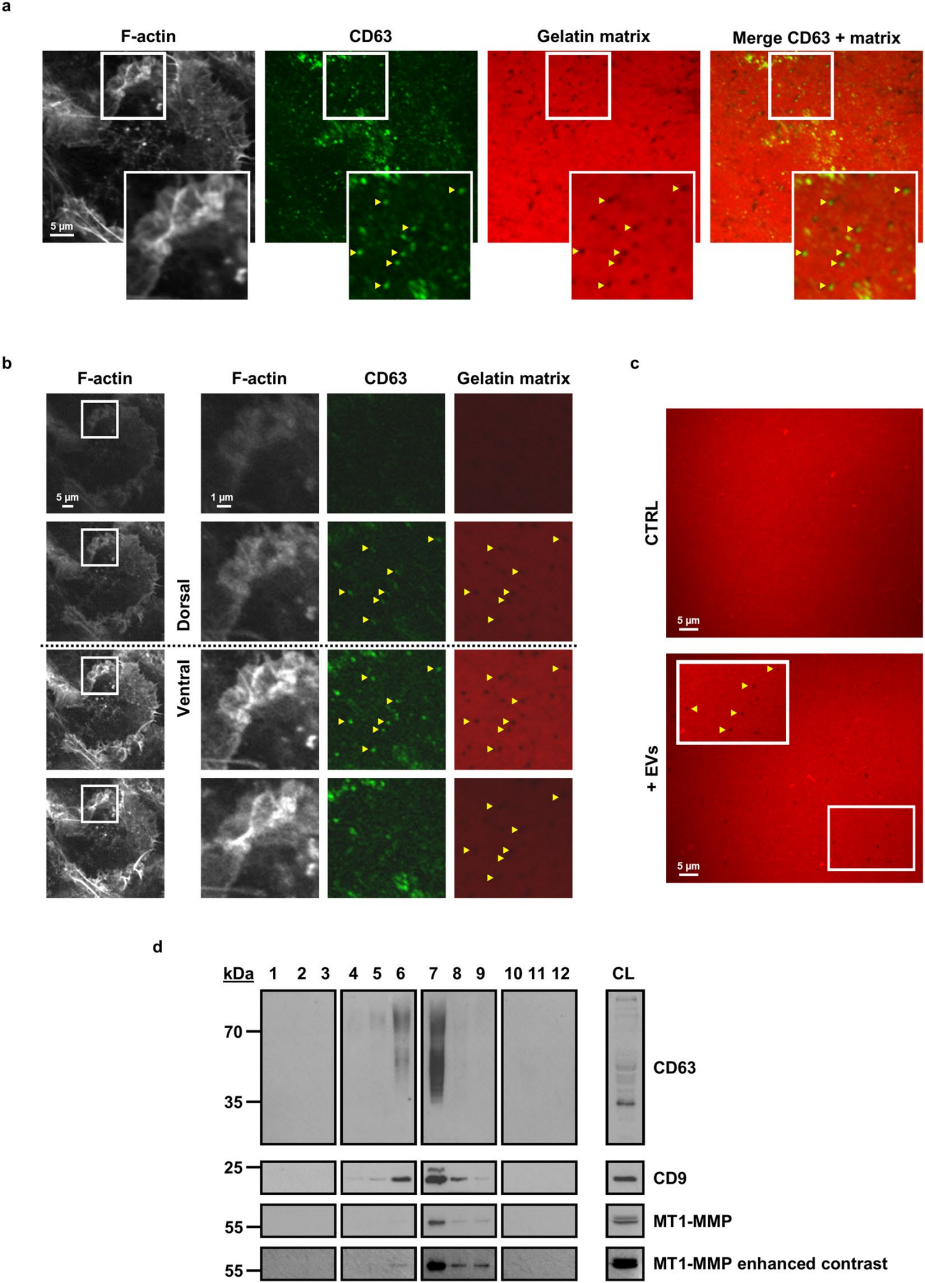
MDA-MB-231 EVs exhibit gelatinolytic activity and contain matrix metalloproteinase MT1-MMP. (**a**) Representative confocal images of parental MDA-MB-231 cells seeded onto a fluorescent gelatin matrix. CD63 colocalizes with areas of matrix degradation, in absence of invadopodium marker F-actin (Acti-Stain 670 phalloidin). Boxed areas are enlarged in the bottom right insets and arrowheads Δ indicate areas of colocalization. (**b**) Z-stacks were taken each 0.5 µm of confocal image in (**a**) in order to localize CD63 in the 3D-space. Highest CD63 signal can be observed at the top surface of the gelatin matrix (third row), whereas the cell cortex signal is visible in all stacks. Boxed areas are enlarged at the right and arrowheads Δ indicate areas of perceptible signal. (**c**) Representative confocal images of fluorescent gelatin matrix without (CTRL) or with EV-incubation (+EVs, 24 h incubation with 4 × 10^10^ EVs). Typical areas of matrix degradation can be observed after incubation with EVs (+EVs), which are completely absent in the control (CTRL) condition. Boxed area is enlarged at the left and arrowheads Δ indicate areas of degradation. (**d**) MT1-MMP is enriched at EV+ fractions of parental MDA-MB-231 breast cancer cells. Representative Western blot after ODG centrifugation shows characteristic enrichment of MT1-MMP at EV+ fractions. CL = crude lysate. A high-contrast blot of MT1-MMP was included to visualize MT1-MMP presence in fraction 6. For reasons of clarity and conciseness, blots were cropped to the bands of interest. Samples derive from the same experiment and gels/blots were processed in parallel. MT1-MMP probings were performed on the same blot as CD9 and flotillin-2, see full-length blots in Supplementary Figure S10.

Mature invadopodia degrade the underlying ECM, achieved by focal delivery of ECM-degrading proteases such as MT1-MMP (MMP-14), MMP-2 and MMP-9^6,42^. However, we occasionally observed dark areas of matrix degradation on a fluorescent gelatin matrix in the absence of invadopodial protein F-actin (phalloidin), but in the presence of CD63-punctae (Fig. 6a). A Z-stack shows maximal CD63 signal intensity at the top surface of the gelatin matrix (Fig. 6b, third row). This prompted us to examine the matrix-degradative capacity of EVs as such. Accordingly, a fluorescent gelatin matrix was incubated with purified EVs and matrix degradation was analysed compared to a PBS control (both diluted with cell culture medium). Typical areas of matrix degradation^43^ can only be observed in the EV-incubated matrix, confirming genuine EV gelatinolytic activity (Fig. 6c and see Supplementary Figure S7). Western blot analysis and shotgun MS indicate that MDA-MB-231 EVs are highly enriched in MT1-MMP (Fig. 6d and see Supplementary Dataset File 2). Although MDA-MB-231 secrete MMP-2 and MMP-9 (see Supplementary Figure S8), these proteases were not detected in EVs by MS (see Supplementary Dataset File 2), suggesting a possible EV-independent way of secretion. Notably, MDA-MB-231 EVs exclusively contain activated MT1-MMP, whereas MDA-MB-231 crude lysate contains both the inactive 64 kDa and active 55 kDa forms (Fig. 6d). No other ECM-degrading metalloproteinases were detected by shotgun MS (see Supplementary Dataset File 2). Hence, we conclude that MDA-MB-231 EVs possess intrinsic gelatinolytic activity, which is probably mediated by the membrane-bound metalloprotease MT1-MMP.

## Discussion

In this study, we have investigated CTTN and FSCN1 function in EV release, using invadopodia-forming MDA-MB-231 breast cancer cells as a model cell line. We therefore combined the high affinity and precise targeting of the nanobody technology with the new gold standard for EV isolation; i.e. ODG centrifugation^34^.

CTTN is a critical factor for MVB docking and thus EV secretion. Rescue experiments using CTTN protein mutants confirmed CTTN-mediated actin branching being essential for EV release^32^. Our results are in line with this, as expression of CTTN NTA Nb2 that is known to decrease ARP2/3-mediated actin polymerization^26^, results in a lower EV release (Fig. 2b). On the other hand, CTTN SH3 domain mutant W525K did not rescue CTTN knock-down effects; excluding a role of this domain in EV release^32^. Our CTTN SH3 Nb2, however, did affect EV release (Fig. 2b). Both nanobody and protein mutant interfere with WIP binding^11,12^, but only the CTTN W525K mutant reduces dynamin-2 binding^12,44^. CTTN SH3 Nb2 possibly interferes with the binding of other CTTN interaction partners involved in EV biogenesis or release, contrary to the protein mutant W525K. In line with this assumption is an observed effect of CTTN SH3 Nb2 expression on LE/lys trafficking and consequently, Golgi morphology (Fig. 3a,b), which was not observed for the SH3-protein mutant^22^.

FSCN1 Nb5 impedes FSCN1’s bundling activity, resulting in thin and loosely packed actin bundles^12^. Expression of this nanobody does not impede EV release (Fig. 2b). However, delocalizing FSCN1 towards the mitochondrial outer membrane using MOM-FSCN1 Nb5^12^ significantly reduces EV release (Fig. 2b). A different effect of delocalizing (MOM-FSCN1 Nb5) versus interfering (FSCN1 Nb5) FSCN1 nanobodies on MMP-9 secretion was previously reported; i.e. not FSCN1 Nb5, but MOM-FSCN1 Nb5, reduces MMP-9 secretion in PC-3 cells^12^. Interestingly, MMP-9 secretion is regulated by microtubules^45^ and researchers demonstrated FSCN1 interacting with microtubules through residues that are not required for F-actin binding. FSCN1 thereby positively regulates microtubule dynamics^46^. Moreover, endosomal transport, which is an essential step in EV biogenesis, occurs largely via microtubules^47^. Hence, we speculate that MOM-FSCN1 Nb5 displaces FSCN1 from microtubules and thus perturbs its regulating function therein. Supportive for this theory is the observed altered Golgi morphology when expressing MOM-FSCN1 Nb5, but not FSCN1 Nb5 (Fig. 3a,b). Besides LE/lys maturation and trafficking, normal Golgi assembly and maintenance depends on both actin and microtubule dynamics^48^. Remarkably, MOM-FSCN1 Nb5 expressing cells have significantly smaller EVs than their non-expressing counterparts (Fig. 2c), which suggests an additional effect on ILV formation.

Strikingly, CK-666 treatment and CTTN shRNA1 expression result in a specific increase of EV release (Fig. 2b and see Supplementary Figure 2), corroborated by a noticeable increase in CD63 intensity on Western blot (see Supplementary Figure 2). These observations were unexpected, as both interventions were previously reported to decrease EV release in head and neck squamous cell carcinoma SCC-61 cells^32^. Although lower CK-666 concentrations were used in the former study, the small-molecule inhibitor is routinely used at 50–100 µM^49^ and we previously optimized CK-666 working concentration for MDA-MB-231 at 100 µM^26^. This working concentration induces detectable defects in invadopodia formation and function, and may however affect mechanisms underlying EV release differently than expected. Of note, the different cell line used in the study may also explain the dissimilar CK-666 concentrations used and/or the different cell response. Nevertheless, caution is warranted with small-molecule inhibitors, as they often act on molecules with pleiotropic functions within the cell and may induce non-EV-related changes^50^. For example, inhibition of neutral sphingomyelinase (i.e. an enzyme involved in EV biogenesis) by means of GW4869 in dendritic cells, surprisingly increases EV release^51^. Up until now, no CK-666 off-target effects have been published, although its sister molecule CK-869 can cause plasma membrane blebbing in ARP2/3-depleted cells^49^. The unexpected increase in EV release induced by CTTN shRNA1 expression may be ascribed to shRNA off-target effects, of which RNAi approaches frequently suffer^52,53^. The fact that we have generated a control scrambled shRNA expressing cell line that non-specifically down-regulates CTTN expression (see Supplementary Figure 1b), further emphasizes the potential promiscuous nature of shRNA. Another possibility is that, for both conditions, the observed increase in EV release results from cellular stress. In this context, André-Grégoire and coworkers recently showed that the chemotherapeutic Temozolomide induces EV release in glioblastoma cells and may even alter EV content, thereby potentially contributing to relapse and aggressive disease^54^. Indeed, tumour cells are known to secrete more EVs than non-transformed cells, probably caused by cellular stress^55^. Accordingly, since CTTN is responsible for serum-independent or growth factor-deprived growth^56^ and MDA-MB-231 were grown in serum-free conditions during EV isolation, knock-down of the protein may induce stress and consequently, an increase in EV release. This also may explain why researchers previously did observe a drop in EV release when expressing CTTN shRNA; cells were grown in growth factor-replete media^32^. Finally, likewise, the ‘chemotherapeutic action’ of high concentration CK-666 may induce cellular stress and a subsequent increase in EV release. This hypothesis is further supported by the fact that both treatments significantly reduced cell numbers in our experimental setup, in contrast to nanobody expression (see Supplementary Figure S3). Hence, our data emphasize the need to exploit different strategies when investigating protein function. Relying on one single method may result in false or incomplete conclusions; the latter was already shown for the actin bundler L-plastin and its role in filopodia extension^57^. In this respect, nanobody technology emerges as a useful complementary strategy.

Besides their (potential) functions in endosomal trafficking, CTTN and FSCN1 both have roles in invadopodia formation and maturation^8,12,26^. Perturbation of these functions could also have repercussions on EV release. Indeed, Hoshino and coworkers showed that invadopodia are critical MVB docking sites and a determining factor for EV release^31^ and we show that MDA-MB-231 do form invadopodial structures on polystyrene plates (Fig. 3c). CTTN SH3 Nb2 and CTTN NTA Nb2 hinder normal invadopodia formation and maturation^12,26^, while both FSCN1 Nb5 and MOM-FSCN1 Nb5 reduce invadopodia stability (and thus lifetime) and maturation^12^. Conspicuously, despite its effect on invadopodia lifetime, FSCN1 Nb5 expression does not alter EV release (Fig. 2b). This suggests that invadopodium longevity is not an absolute requirement for EV release. Another possibility is that invadopodia are not a prerequisite for EV release. This would mean that all other invadopodium markers tested by others and us (TKS5, N-WASP, PI3K^31^ and CTTN) have additional roles in endosomal trafficking, which would then completely account for the observed effects on EV release. In this respect, it would be interesting to investigate whether targeting of some ‘invadopodium markers’ in non-invadopodia forming cells, such as MCF-7, impacts EV release. Worth mentioning here is that RAB27B-overexpressing MCF-7, a cancer cell model that secretes high amounts of EVs^30,34^, do not suddenly form invadopodia-like protrusions on gelatin matrix (See Supplementary Figure S9). However, it seems that invadopodia-forming cells preferentially target their MVBs towards these ventral protrusions for delivery, as MVBs and invadopodia are closely associated (Fig. 4 and^31^). Furhermore, when ABP nanobody expression induces invadopodia dispersion, the MVBs seem to ‘follow’ this pattern change (Fig. 5 and see Supplementary Figure S6).

Recently, Havrylov and Park postulated that EVs are bona fide invasion-associated structures of cancer cells, similar to invadopodia and other specialised protrusive cellular structures^4^. EVs contribute to the processes of invasion and metastasis in many ways^3^. Moreover, there is a strong connection between the invadopodial and EV proteome^4^. Indeed, we also observed many proteins in MDA-MB-231 EVs that are classified as common invadopodial proteins or proteins involved in invadopodium assembly and maturation. These include signalling molecules (EGFR^58^, TGFR, Src^59^, CDC42^60^, RhoA^61^ and RhoC^62^), proteins involved in invadopodium precursor formation (cofilin-1^62^, several subunits of the ARP2/3 complex^60^, FSCN1^12^, α-actinin-1/4^63^ and actin^64^), invadopodium function (IQGAP1, VAMP7^61^, dynamin-2^65^, MT1-MMP^61^, tubulins^64^, integrins^66^, talin, ezrin, radixin, moesin^67^, α-actinin-1/4^63^ and vimentin^64^) and invadopodium adhesion to the ECM (vinculin, paxillin^59^, filamin^68^ and integrin^66^). Unexpectedly, we did not detect key invadopodium markers CTTN, TKS5 and N-WASP^7^ in MDA-MB-231 EVs (see Supplementary Dataset File 2). Metabolic enzymes GAPDH, PKM, LDHA and G6PD have also been detected in invadopodia^4^ and are, with the exception of G6PD, included in the ExoCarta Top 100 list (see http://exocarta.org/). These glycolytic enzymes are also highly abundant in our purified EVs (see Supplementary Dataset File 2). Considering this invadopodial cargo, it is quite conceivable that auto- or paracrine EV uptake could enhance invadopodia formation and function, as was seen by Hoshino and coworkers^31^. It also could (in part) explain the observed positive feedback loop between LE/lys fusion of MT1-MMP vesicles and invadopodia formation and function^69^. Anyhow, our proteomics data further corroborate a close interconnection between invadopodia and EVs.

Analysis of EV proteome isolated from stable MDA-MB-231 cells showed a potential contribution of the CTTN SH3 domain and FSCN1 in EV protein sorting. CD99L2-sorting could be negatively regulated by the CTTN SH3 domain, whereas FSCN1, again independently of its actin bundling activity, possibly promotes sorting of RPL18 (See Supplementary Figure S4 and Supplementary Dataset File 3). Importantly, due to the relatively small EV proteome compared to for instance whole cell proteome, analysis of a larger number of samples is needed in order to perform rational statistical analysis and thus, is needed to sort out whether the observed differences are statistically significant or not. Sinha and colleagues identified other proteins as potentially different cargoes between CTTN knocked-down and control EVs of SCC-61 cells. However, at least two out of the 22 candidate proteins were false positives^32^. Hence, more research is required to investigate the role of CTTN and FSCN1 in determining EV cargo composition.

We observed MVBs colocalizing with areas of matrix degradation, in absence of invadopodium marker F-actin (Fig. 6a,b). Limited by confocal microscopy resolution, it is however not possible to pin-point the exact location of the CD63 signal (Fig. 6b; the cell cortex signal is visible in all stacks). The signal may localize extracellularly and hence originate from secreted EVs that were left behind post-assembly of the invadopodium. On the other hand, the CD63 signal may localize intracellularly and thus may originate from MVBs. In the latter case, it is possible that MVB recruitment precedes invadopodium formation or that MVBs transiently remain after invadopodium retraction. Another possibility is that MVB docking and secretion also occur in absence of invadopodia. Nevertheless, we show that MDA-MB-231 EVs possess an intrinsic ability for proteolytic degradation of the ECM, which is probably mediated by MT1-MMP as MDA-MB-231 EVs are enriched with the membrane-bound metalloproteinase (Fig. 6d and see Supplementary Dataset File 2). MT1-MMP has already been identified in EV cargo of other cell lines^31,32,70^, whether or not together with MMP-2^31,32^ and MMP-9^32^. Intriguingly, although MS did not detect MMP-2 and MMP-9 in EVs (see Supplementary Dataset File 2), MDA-MB-231 do secrete both proteins (Supplementary Figure S8). This may indicate a specific EV sorting mechanism for metalloproteinase MT1-MMP, yet additional research is warranted to confirm this. MT1-MMP not only degrades ECM, but also assists in activating other MMPs such as MMP-2. In this respect, Hakulinen and coworkers demonstrated that COS-1 cells-derived pro-MMP-2 could be activated by EVs purified from MT1-MMP overexpressing HT-1080 cells^70^. These and our results suggest that EV-associated MT1-MMP could promote auto- or paracrine MMP-2 activation, the latter possibly being secreted in an EV-independent way (see Supplementary Figure S8). Of note, MT1-MMP is known to be specifically targeted to invadopodia^6,42^, again supportive for an interconnection between invadopodia and EVs. Thus, we hypothesize that EV secretion is a way of redirecting MT1-MMP activity away from the plasma membrane. Like this, cancer cells further stimulate pericellular matrix degradation in concert with plasma membrane-bound and secreted MMPs.

**Figure 7:**
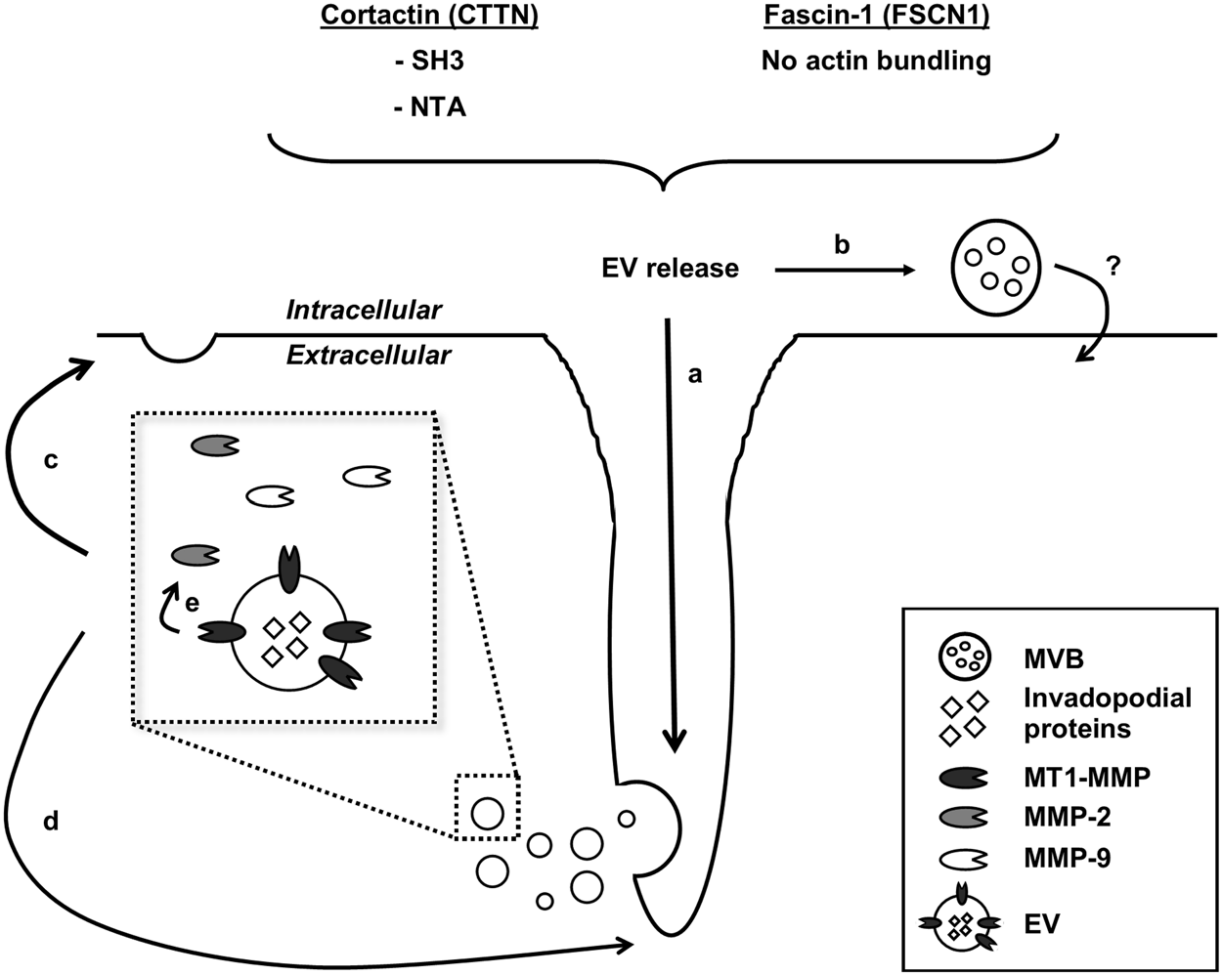
Model summarizing the obtained data. Both CTTN SH3 and NTA domain positively regulate EV release in invadopodia-forming MDA-MB-231. FSCN1 stimulates EV release independent of its actin-bundling function. EVs are preferentially secreted at invadopodia (**a**), but we cannot rule out the existence of other MVB docking sites (**b**). EVs are enriched in invadopodial proteins and auto- or paracrine EV uptake presumably stimulates *de novo* invadopodia formation (**c**) or stimulates existing invadopodia lifetime and maturation (**d**). Finally, EV possess matrix-degradative capacity, likely mediated by MT1-MMP, as the EVs are enriched in the membrane-bound metalloproteinase. MMP-9 and MMP-2 are potentially secreted in an EV-independent way and the latter MMPs may be activated by MT1-MMP (**e**). The concerted action of all MMPs is responsible pericellular matrix degradation and contributes to the overall invasive behaviour of MDA-MB-231 breast cancer cells.

In conclusion, we demonstrate that the CTTN NTA domain, and thus CTTN-regulated branched actin assembly, contributes to EV release. We reveal a role of the CTTN SH3 domain in EV release which, up until now, remained unnoticed by routine use of the W525K mutant. Furthermore, we show for the first time that EV release occurs independently of FSCN1’s F-actin bundling activity and presumably depends on FSCN1’s microtubule-regulating function. Whether the observed nanobody effects are the result of a perturbed endosomal trafficking, invadopodia formation/maturation or both, is still unclear. However, we confirm that MVBs spatially organize near invadopodia and that EVs are enriched with invadopodial proteins. Moreover, EVs possess genuine gelatinolytic activity, the latter presumably mediated by invadopodia marker MT1-MMP. Hence, our data confirm recently reported association between invadopodia and EV release (see summarizing model in Fig. 7), but also highlights the importance of discerning bona fide EV-regulating from invadopodia-regulating protein functions in future experiments. We believe that nanobodies can play an important role therein, as they can impinge on protein functionality with surgical precision.

## Methods

### Generation of ABP nanobodies

See Supplementary Methods for details on the generation of ABP-specific nanobodies

### Antibodies, reagents and buffer preparations

See Supplementary Methods for a detailed description on the antibodies, reagents and buffer preparations used in this study.

### cDNA cloning

See Supplementary Methods for a detailed description on the cloning reactions.

### Cell culture and transduction

See Supplementary Methods for a detailed description on the cell lines, culture conditions and transduction.

### Validation of stable shRNA MDA-MB-231

See Supplementary Methods for details on the validation of MDA-MB-231 stably expressing shRNA.

### Assessment of cell numbers and viability

See Supplementary Methods for a detailed description on the assessment of cell numbers and viability.

### EV isolation using ODG centrifugation

A brief overview of the protocol is shown in Fig. 1a. A discontinuous OptiPrep gradient was used to isolate EVs from MDA-MB-231 (described in^34^, with some adjustments). In detail, cells were cultured in 5 x T175 flasks until a density of ±80% was reached (day 1). Cells were washed three times using serum-free medium and were further grown on serum-free medium. Nanobody expression was induced by adding 500 ng/ml doxycycline (+dox, 5× T175) and these were grown in parallel with non-induced cells (−dox, 5× T175 without nanobody expression). 24 h post-medium change, CM was collected and cells were placed on new serum-free medium (− or +dox) for another 24 h (day 2). On day 3, CM was again collected and cells were counted using a Bürker chamber. In order to eliminate dead cells, debris and large vesicles, the CM was centrifuged (3 min at 75 g using a swinging bucket rotor) and filtered (0.22 µm and 0.45 µm). Next, the CM was concentrated via ultrafiltration using Amicon Ultra-15 10 K regenerated cellulose filters (Merck, Billerica, Massachusetts, USA) (4,000 g at 4 °C, spin times of 20 min using a swinging bucket rotor) until a volume of < 500 µl was reached. Subsequently, a discontinuous OptiPrep gradient was prepared by layering 3 ml of 40%, 3 ml of 20%, 3 ml of 10% and 2.5 ml of 5% OptiPrep gradient solutions (see section Antibodies, reagents and buffer preparations in Supplementary Methods) in a 13.2 ml open top polypropylene tube (Beckman Coulter, Fullerton, California, USA). The concentrated CM was placed on top of the discontinuous gradient, followed by 18 h ultracentrifugation at 100,000 g and 4 °C using SW41 Ti rotor (Beckman Coulter). Fractions of 1 ml were collected and diluted with 10 ml PBS (without Ca^2+^ and Mg^2+^) in 13.2 ml open top polypropylene tubes, followed by ultracentrifugation (3 h at 100,000 g and 4 °C using SW41 Ti rotor) in order to pellet the EVs (day 4). The polypropylene tubes were quickly decanted and put upright to drain the PBS. Finally, the invisible EV pellet was resuspended in 50 µl fresh PBS (without Ca^2+^ and Mg^2+^) and stored at −80 °C until further use.

For isolation of CTTN shRNA1 MDA-MB-231 EVs, cells were incubated with 500 ng/ml dox 72 h prior medium change (day 1) and were further grown on dox to support shRNA expression. The ARP2/3-inhibitor CK-666 or DMSO (control) were added to a final concentration of 100 µM on day 1 and day 2, replacing dox addition.

We have submitted all relevant data of our experiments to the EV-TRACK knowledgebase (EV-TRACK ID: EV180017, http://evtrack.org)^71^.

### EV characterization

#### EV density

See Supplementary Methods for details on EV density determination.

#### EV protein analysis by Western blot

See Supplementary Methods for details on EV protein analysis by Western blot.

#### EV concentration and mean size

Following EV isolation, fractions were tested for presence of EV-markers CD63 and CD9 by Western blot. Mean concentration and size (with corresponding standard error) of particles found in these EV-positive (EV+) fractions were determined by means of nanoparticle tracking analysis and each EV+ fraction was measured separately. Nanoparticle tracking analysis was performed using a NanoSight LM10 microscope (Malvern Instruments Ltd, Malvern, UK) equipped with a 488 nm laser and with temperature monitoring. For each EV+ fraction, three 30 second videos were recorded and analysed with nanoparticle tracking analysis software version 3.2 (camera level 13 and threshold detection 3). PBS (without Ca^2+^ and Mg^2+^) was used to dilute EV-fractions within measuring range of the NanoSight (3 × 10^8^ − 1 × 10^9^ particles/ml).

At least 3 biological replicates (n ≥ 3) each cell line were characterized using nanoparticle tracking analysis. A biological replicate consists of an induced (+dox/CK-666) versus non-induced (−dox/DMSO) cell line run in parallel, i.e. same passage number of which EVs were isolated and characterized in parallel. Mean EV concentration of each replicate was expressed as the number of particles per cell, taking all EV+ fractions and cell number into account. Seeing nanobody expression has a multiplicative effect on EV release, mean concentration data were transformed to their logarithms (base 10) and analysed using a ratio paired t test (−dox/DMSO versus + dox/CK-666 replicates of a single cell line, n ≥ 3, α = 0.05, two-tailed, Shapiro-Wilk normality test passed) and using GraphPad Prism (GraphPad Software, La Jolla, CA, USA). For vesicle size assessment, mean particle size of all isolated EV+ fractions was plotted and analysed by means of a Mann-Whitney rank sum test (ordinal and independent observations which are not normally distributed; n ≥ 6, α = 0.05, two-tailed), comparing−dox/DMSO versus + dox/CK-666 of a single cell line (identical colours on the graph).

#### EV protein analysis by shotgun MS

See Supplementary Methods for details on the shotgun MS experiment.

### Immunofluorescence and microscopy

Coverslips were coated with 0.01% gelatin in PBS (1 h at room temperature), fixed with 0.5% glutaraldehyde (10 min on ice) and quenched with medium (1 h at 37 °C). For visualisation of matrix degradation, coverslips were coated with Cy3-gelatin using the QCM Gelatin Invadopodia Assay (Merck) and according to the manufacturer’s protocol. Cells were seeded at a density of ±60–80% onto coated coverslips or in 24-well polystyrene plates. If desired, nanobody expression was induced by adding dox to a final concentration of 500 ng/ml post-cell adhesion. To investigate EV-mediated matrix degradation, Cy3-gelatin matrices were incubated with equal volumes of PBS (without Ca^2+^ and Mg^2+^, control, n = 3) or EVs in PBS (n = 3), both diluted with medium supplemented with 10% fetal bovine serum. EVs were diluted to a final concentration of 4 × 10^10^ EVs each coverslip. Immunostaining was performed 24 h later as previously described^27^. Briefly, cells were fixed with 3% paraformaldehyde for 25 min, permeabilized during 5 min with 0.2% Triton X-100 and additionally incubated with 0.75% glycine for 20 min. Every incubation step was followed by at least three wash steps (PBS with Ca^2+^ and Mg^2+^). After a blocking step with 1% bovine serum albumin (BSA), coverslips were incubated with primary and secondary antibodies (1 h at 37 °C and 30 min at room temperature, respectively), with both antibodies diluted in 1% BSA. Nuclei and F-actin were stained during the last antibody incubation step with DAPI and phalloidin, respectively. Antibody incubation steps were separated by four 4 min wash steps in 1% BSA. Finally, cells were washed twice with PBS. Coverslips were mounted onto microscope slides using Vectashield antifade mounting medium (Vector Laboratories, Burlingame, CA, USA) and were sealed with nail polish. Cells were analysed at room temperature using a Zeiss Axiovert 200 M fluorescence microscope with Apotome module (Zeiss 63 × 1.4-NA Oil Plan-Apochromat objective, Carl Zeiss, Oberkochen, Germany) with Axiovision 4.5 software (Zeiss), an Olympus IX81 Fluoview 1000 confocal laser scanning microscope (Olympus x60 1.35-NA Oil UPLSAPO objective, Olympus, Tokyo, Japan) with FluoView FV 1000 software (Olympus) or Olympus IX71 inverted fluorescence and phase contrast microscope (Olympus 10 × 2 0.3-NA UPLFLN phase, 20 × 0.45-LUCPLFLN phase objective, Olympus) with Cell^B^ software (Olympus). Z-stacks were taken each 0.5 µm with the Olympus confocal scanning microscope.

Microscopy images were analysed by means of ImageJ (National Institutes of Health, Bethesda, MD, USA). Golgin-245, CD63 and phalloidin (i.e. invadopodia and cell cortex) spread area were marked out using the freehand selection tool. Surface area of selected areas was determined using following macro: run (“Create Mask”); run (“Analyze Particles…”, “size = 0-Infinity circularity = 0.00–1.00 show = Outlines display clear”). For CD63 dot quantification, images were converted to 8-bit (run (“8-bit”)) and background was subtracted (run (“Subtract Background…”, “rolling = 50”)). Next, the 8-bit images were binarised using Li’s Minimum Cross Entropy thresholding method (set Auto Threshold (“Li dark”); set Threshold (18,255)), followed by classic watershed segmentation (run (“Watershed”)). CD63 dot quantification was finally achieved using run (“Analyze Particles…”, “size = 0-Infinity circularity = 0.00–1.00 show = Outlines display clear”). A Mann-Whitney rank sum test (ordinal and independent observations which are not normally distributed; n ≥ 100 split up into ≥ 3 independent experiments, α = 0.05, two-tailed) comparing induced (+dox) to non-induced (−dox) of a single cell line (identical colours on the graph), comparing invadopodia area to total cell area or comparing invadopodia area to total cell area without the invadopodia region, was used to analyse the results via Graphpad.

### Gelatin zymography

See Supplementary Methods for a description on the gelatin zymography.

## Electronic supplementary material

Supplementary Information

Supplementary Dataset File 1

Supplementary Dataset File 2

Supplementary Dataset File 3

## Data Availability

All data generated or analysed during this study are included in this published article (and its Supplementary Information files). Nanobody sequences are licensed to Gulliver Biomed.

## References

[1] Tkach M, Thery C (2016). Communication by Extracellular Vesicles: Where We Are and Where We Need to Go. Cell.

[2] Kowal J, Tkach M, Thery C (2014). Biogenesis and secretion of exosomes. Curr. Opin. Cell Biol..

[3] Hendrix A, Hume AN (2011). Exosome signaling in mammary gland development and cancer. Int J Dev Biol.

[4] Havrylov S, Park M (2015). MS/MS-based strategies for proteomic profiling of invasive cell structures. Proteomics.

[5] Yamaguchi H (2012). Pathological roles of invadopodia in cancer invasion and metastasis. Eur. J. Cell Biol..

[6] Poincloux R, Lizarraga F, Chavrier P (2009). Matrix invasion by tumour cells: a focus on MT1-MMP trafficking to invadopodia. J. Cell Sci..

[7] Beaty BT, Condeelis J (2014). Digging a little deeper: The stages of invadopodium formation and maturation. Eur. J. Cell Biol..

[8] Kirkbride KC, Sung BH, Sinha S, Weaver AM (2011). Cortactin: a multifunctional regulator of cellular invasiveness. Cell Adh Migr.

[9] McNiven MA (2000). Regulated interactions between dynamin and the actin-binding protein cortactin modulate cell shape. The Journal of cell biology.

[10] Mizutani K, Miki H, He H, Maruta H, Takenawa T (2002). Essential role of neural Wiskott-Aldrich syndrome protein in podosome formation and degradation of extracellular matrix in src-transformed fibroblasts. Cancer Res.

[11] Kinley AW (2003). Cortactin interacts with WIP in regulating Arp2/3 activation and membrane protrusion. Current biology: CB.

[12] Van Audenhove I (2014). Stratifying fascin and cortactin function in invadopodium formation using inhibitory nanobodies and targeted subcellular delocalization. FASEB journal: official publication of the Federation of American Societies for Experimental Biology.

[13] Cao H (2003). Cortactin is a component of clathrin-coated pits and participates in receptor-mediated endocytosis. Mol. Cell. Biol..

[14] Zhu JW (2005). Regulation of cortactin/dynamin interaction by actin polymerization during the fission of clathrin-coated pits. J. Cell Sci..

[15] Sauvonnet N, Dujeancourt A, Dautry-Varsat A (2005). Cortactin and dynamin are required for the clathrinin-dependent endocytosis of gamma c cytokine receptor. J. Cell Biol..

[16] Puthenveedu MA (2010). Sequence-Dependent Sorting of Recycling Proteins by Actin-Stabilized Endosomal Microdomains. Cell.

[17] Cao H, Chen J, Krueger EW, McNiven MA (2010). Src-Mediated Phosphorylation of Dynamin and Cortactin Regulates the “Constitutive” Endocytosis of Transferrin. Mol. Cell. Biol..

[18] Grassart A (2010). Pak1 Phosphorylation Enhances Cortactin-N-WASP Interaction in Clathrin-Caveolin-Independent Endocytosis. Traffic.

[19] Sung BH, Zhu XD, Kaverina I, Weaver AM (2011). Cortactin Controls Cell Motility and Lamellipodial Dynamics by Regulating ECM Secretion. Curr. Biol..

[20] Ohashi E (2011). Receptor Sorting within Endosomal Trafficking Pathway Is Facilitated by Dynamic Actin Filaments. PLoS One.

[21] Hong NH, Qi AD, Weaver AM (2015). PI(3,5)P-2 controls endosomal branched actin dynamics by regulating cortactin-actin interactions. J. Cell Biol..

[22] Kirkbride KC (2012). Regulation of late endosomal/lysosomal maturation and trafficking by cortactin affects Golgi morphology. Cytoskeleton.

[23] Courson DS, Rock RSAC-link (2010). Assembly and Disassembly Mechanics for alpha-Actinin and Fascin. J. Biol. Chem..

[24] Sedeh RS (2010). Structure, Evolutionary Conservation, and Conformational Dynamics of Homo sapiens Fascin-1, an F-actin Crosslinking Protein. Journal of molecular biology.

[25] Yang SY (2013). Molecular Mechanism of Fascin Function in Filopodial Formation. J. Biol. Chem..

[26] Bertier Laurence, Boucherie Ciska, Zwaenepoel Olivier, Vanloo Berlinda, Van Troys Marleen, Van Audenhove Isabel, Gettemans Jan (2017). Inhibitory cortactin nanobodies delineate the role of NTA- and SH3-domain–specific functions during invadopodium formation and cancer cell invasion. The FASEB Journal.

[27] Beghein E (2016). A new survivin tracer tracks, delocalizes and captures endogenous survivin at different subcellular locations and in distinct organelles. Sci Rep.

[28] Beghein E, Gettemans J (2017). Nanobody Technology: A Versatile Toolkit for Microscopic Imaging, Protein-Protein Interaction Analysis, and Protein Function Exploration. Front Immunol.

[29] Van Audenhove Isabel, Denert Majken, Boucherie Ciska, Pieters Leen, Cornelissen Maria, Gettemans Jan (2016). Fascin Rigidity and L-plastin Flexibility Cooperate in Cancer Cell Invadopodia and Filopodia. Journal of Biological Chemistry.

[30] Ostrowski M (2010). Rab27a and Rab27b control different steps of the exosome secretion pathway. Nat. Cell Biol..

[31] Hoshino D (2013). Exosome Secretion Is Enhanced by Invadopodia and Drives Invasive Behavior. Cell Reports.

[32] Sinha S (2016). Cortactin promotes exosome secretion by controlling branched actin dynamics. The Journal of cell biology.

[33] Ji H (2013). Proteome profiling of exosomes derived from human primary and metastatic colorectal cancer cells reveal differential expression of key metastatic factors and signal transduction components. Proteomics.

[34] Van Deun, J. *et al*. The impact of disparate isolation methods for extracellular vesicles on downstream RNA profiling. *J Extracell Vesicles* 3, 10.3402/jev.v3403.24858 (2014). 10.3402/jev.v3.24858 PMC4169610 25317274

[35] Vergauwen G (2017). Confounding factors of ultrafiltration and protein analysis in extracellular vesicle research. Sci Rep.

[36] Villarreal L (2013). Unconventional secretion is a major contributor of cancer cell line secretomes. Molecular & cellular proteomics: MCP.

[37] Van den Abbeele A (2010). A llama-derived gelsolin single-domain antibody blocks gelsolin-G-actin interaction. Cellular and molecular life sciences: CMLS.

[38] Pollard TD (2007). Regulation of actin filament assembly by Arp2/3 complex and formins. Annu. Rev. Biophys. Biomolec. Struct..

[39] Merrifield CJ, Qualmann B, Kessels MM, Almers W (2004). Neural Wiskott Aldrich Syndrome Protein (N-WASP) and the Arp2/3 complex are recruited to sites of clathrin-mediated endocytosis in cultured fibroblasts. Eur. J. Cell Biol..

[40] Daugherty KM, Goode BL (2008). Functional surfaces on the p35/ARPC2 subunit of Arp2/3 complex required for cell growth, actin nucleation, and endocytosis. J. Biol. Chem..

[41] Basquin C (2015). Membrane protrusion powers clathrin-independent endocytosis of interleukin-2 receptor. Embo J..

[42] Stylli SS, Kaye AH, Lock P (2008). Invadopodia: At the cutting edge of tumour invasion. J. Clin. Neurosci..

[43] Clancy JW (2015). Regulated delivery of molecular cargo to invasive tumour-derived microvesicles. Nat. Commun..

[44] Schafer DA (2002). Dynamin2 and cortactin regulate actin assembly and filament organization. Curr. Biol..

[45] Schnaeker EM (2004). Microtubule-dependent matrix metalloproteinase-2/matrix metalloproteinase-9 exocytosis: Prerequisite in human melanoma cell invasion. Cancer Res..

[46] Villari G (2015). A direct interaction between fascin and microtubules contributes to adhesion dynamics and cell migration. J. Cell Sci..

[47] Huotari J, Helenius A (2011). Endosome maturation. Embo J..

[48] Copeland SJ, Thurston SF, Copeland JW (2016). Actin- and microtubule-dependent regulation of Golgi morphology by FHDC1. Molecular biology of the cell.

[49] Rotty JD, Wu CY, Bear JE (2013). New insights into the regulation and cellular functions of the ARP2/3 complex. Nat. Rev. Mol. Cell Biol..

[50] Bobrie A, Colombo M, Raposo G, Thery C (2011). Exosome secretion: molecular mechanisms and roles in immune responses. Traffic.

[51] Colombo, M., Raposo, G. & Thery, C. In *Annual Review of Cell and Developmental Biology, Vol 30* Vol. 30 *Annual Review of Cell and Developmental Biology* (eds Schekman, R. & Lehmann, R.) 255–289 (2014). 10.1146/annurev-cellbio-101512-122326 25288114

[52] Jackson AL, Linsley PS (2010). Recognizing and avoiding siRNA off-target effects for target identification and therapeutic application. Nat. Rev. Drug Discov..

[53] Renicke C, Schuster D, Usherenko S, Essen LO, Taxis C (2013). A LOV2 Domain-Based Optogenetic Tool to Control Protein Degradation and Cellular Function. Chem. Biol..

[54] Andre-Gregoire, G., Bidere, N. & Gavard, J. Temozolomide affects Extracellular Vesicles Released by Glioblastoma Cells. *Biochimie*, 10.1016/j.biochi.2018.02.007 (2018). 10.1016/j.biochi.2018.02.007 29454008

[55] Whiteside, T. L. In *Advances in Clinical Chemistry* Vol. 74 (ed. Makowski, G. S.) 103–141 (Elsevier Academic Press Inc, 2016).

[56] Clark ES (2009). Aggressiveness of HNSCC tumors depends on expression levels of cortactin, a gene in the 11q13 amplicon. Oncogene.

[57] Delanote V (2010). An alpaca single-domain antibody blocks filopodia formation by obstructing L-plastin-mediated F-actin bundling. FASEB journal: official publication of the Federation of American Societies for Experimental Biology.

[58] Zhou ZN (2014). Autocrine HBEGF expression promotes breast cancer intravasation, metastasis and macrophage-independent invasion *in vivo*. Oncogene.

[59] Pignatelli J, Tumbarello DA, Schmidt RP, Turner CE (2012). Hic-5 promotes invadopodia formation and invasion during TGF-beta-induced epithelial-mesenchymal transition. J. Cell Biol..

[60] Yamaguchi H (2005). Molecular mechanisms of invadopodium formation: the role of the N-WASP-Arp2/3 complex pathway and cofilin. J. Cell Biol..

[61] Sakurai-Yageta M (2008). The interaction of IQGAP1 with the exocyst complex is required for tumor cell invasion downstream of Cdc42 and RhoA. J. Cell Biol..

[62] Bravo-Cordero JJ (2011). A Novel Spatiotemporal RhoC Activation Pathway Locally Regulates Cofilin Activity at Invadopodia. Curr. Biol..

[63] Yamaguchi H (2017). Actinin-1 and actinin-4 play essential but distinct roles in invadopodia formation by carcinoma cells. Eur. J. Cell Biol..

[64] Schoumacher M, Goldman RD, Louvard D, Vignjevic DM (2010). Actin, microtubules, and vimentin intermediate filaments cooperate for elongation of invadopodia. J. Cell Biol..

[65] Zhang YB (2016). Dynamin2 GTPase contributes to invadopodia formation in invasive bladder cancer cells. Biochem. Biophys. Res. Commun..

[66] Mueller SC (1999). A novel protease-docking function of integrin at invadopodia. J. Biol. Chem..

[67] Beaty BT (2014). Talin regulates moesin-NHE-1 recruitment to invadopodia and promotes mammary tumor metastasis. J. Cell Biol..

[68] Takkunen M, Hukkanen M, Liljestrom M, Grenman R, Virtanen I (2010). Podosome-like structures of non-invasive carcinoma cells are replaced in epithelial-mesenchymal transition by actin comet-embedded invadopodia. J. Cell. Mol. Med..

[69] Steffen A (2008). MT1-MMP-dependent invasion is regulated by TI-VAMP/VAMP7. Curr. Biol..

[70] Hakulinen J, Sankkila L, Sugiyama N, Lehti K, Keski-Oja J (2008). Secretion of Active Membrane Type 1 Matrix Metalloproteinase (MMP-14) Into Extracellular Space in Microvesicular Exosomes. J. Cell. Biochem..

[71] Consortium E-T (2017). EV-TRACK: transparent reporting and centralizing knowledge in extracellular vesicle research. Nat Methods.

