# Supplementary material for: Cortactin and fascin-1 regulate extracellular vesicle release by controlling endosomal trafficking or invadopodia formation and function
Source: Sci Rep. 2018 Oct 23;8:15606. doi: 10.1038/s41598-018-33868-z (PMC6199335; doi:10.1038/s41598-018-33868-z)
Supplement: Supplementary file 1 — Supplementary Information [file 41598_2018_33868_MOESM1_ESM.pdf]

CORTACTIN AND FASCIN-1 REGULATE EXTRACELLULAR VESICLE RELEASE BY CONTROLLING ENDOSOMAL TRAFFICKING OR INVADOPODIA FORMATION AND FUNCTION

Authors

Els Beghein[1], Delphine Devriese[1], Evy Van Hoey[1] and Jan Gettemans*[1]

Affiliations

[1]Department of Biomolecular Medicine, Faculty of Medicine and Health Sciences, Campus Rommelaere, A. Baertsoenkaai 3, Ghent University, Ghent, Belgium.

[*]Corresponding author:
Department of Biomolecular Medicine,
Faculty of Medicine and Health Sciences,
Campus Rommelaere, Ghent University
Albert Baertsoenkaai 3
B-9000 Ghent, Belgium
Tel: + 32 9 2649340
Fax: + 32 9 2649490
E-mail: jan.gettemans@ugent.be

SUPPLEMENTARY DATASETS

Supplementary Dataset File 1 (See additional .xlsx file)

Supplementary Dataset File 2 (See additional .xlsx file)

Supplementary Dataset File 3 (See additional .xlsx file)

SUPPLEMENTARY FIGURES

Supplementary Figure S1

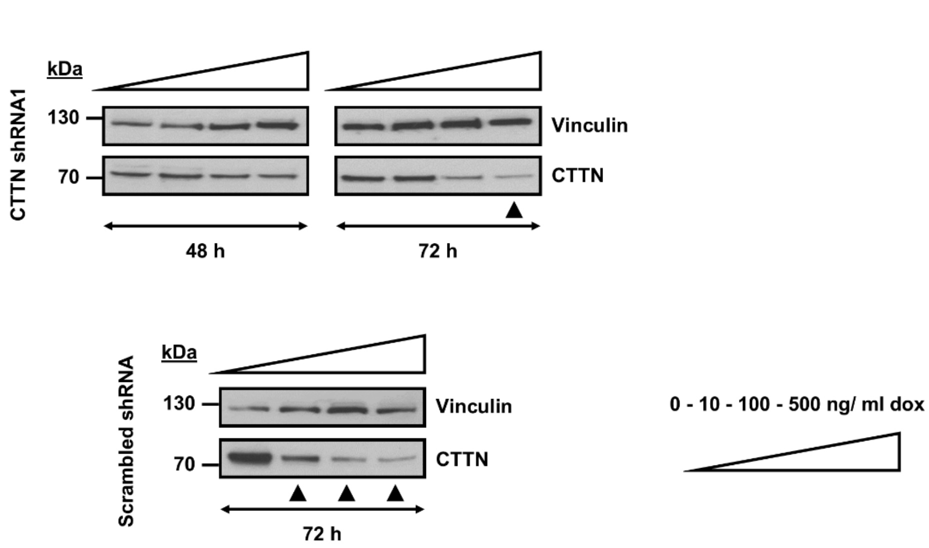

Supplementary Figure S1. Validation of stable MDA-MB-231 cells with dox-inducible expression of scrambled shRNA or shRNA targeting CTTN expression. (a) Determination of endogenous CTTN protein levels in the generated stable CTTN shRNA1 cell line after induction with different concentrations of dox during 48 h or 72 h. 72 h incubation with 500 ng/ ml dox reduces CTTN protein levels on Western blot (arrowhead △) with ± 80 % compared to its 72 h non-induced counterpart. Vinculin was used as a loading control and protein levels were estimated by using ImageJ software. **(b)** Determination of endogenous CTTN protein levels in the generated stable scrambled shRNA cell line after induction with different concentrations of dox during 72 h. CTTN expression diminishes steadily when incubating with higher dox concentrations (arrowheads △). Vinculin was used as a loading control. For reasons of clarity and conciseness, blots were cropped to the bands of interest. Full-length blots are presented in Supplementary Figure S10.

Supplementary Figure S2

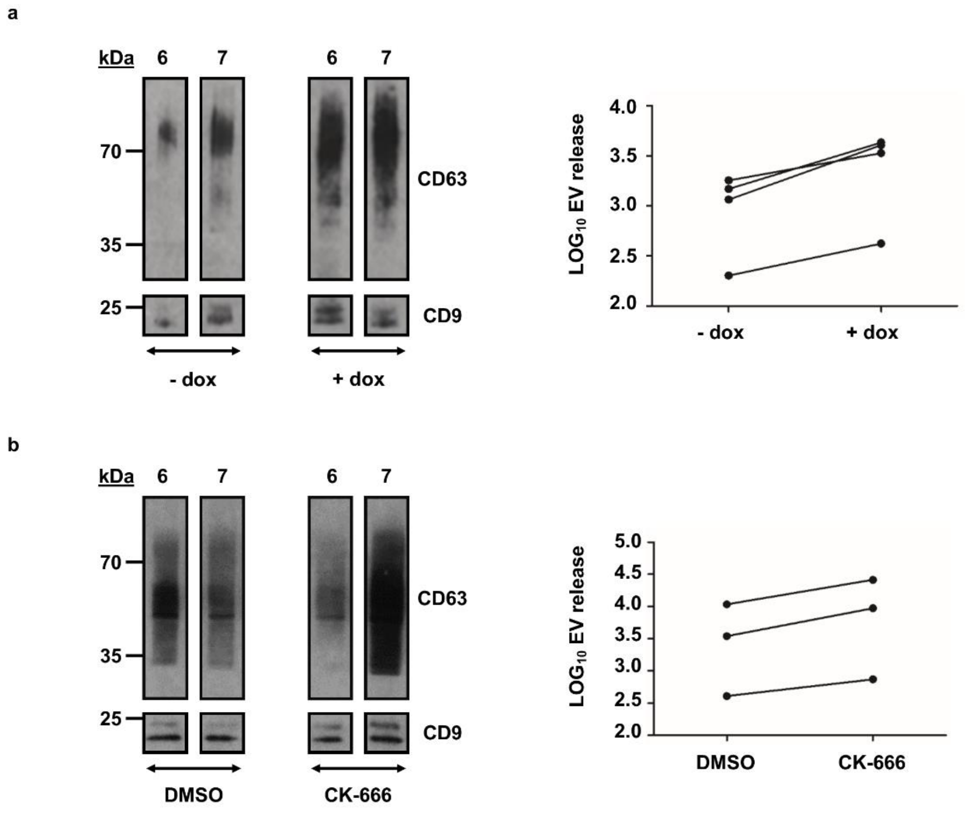

Supplementary Figure S2. Expression of CTTN shRNA1 or incubation with ARP2/3 inhibitor CK-666 induces an increase in EV release. (a) EVs of CTTN shRNA1 expressing (+ dox, 72 h incubation with 500 ng/ ml dox) or non-expressing (- dox) MDA-MB-231 cells were isolated and quantified by nanoparticle tracking analysis. Representative Western blots on the left of EV+ fractions 6 and 7 show an increase in EV release when CTTN shRNA1 expression is induced. Graphs on the right represent LOG EV release of each biological replication (n = 4) and corroborate the Western blot data. **(b)** Parental MDA-MB-231 breast cancer cells were incubated with 100 µM CK-666 during 24 h prior to EV isolation and quantification by nanoparticle tracking analysis. Representative Western blots on the left of EV+ fractions 6 and 7 show an increase in EV release when incubated with CK-666. Graphs on the right represent LOG EV release of each biological replication (n = 3) and corroborate the Western blot data. For reasons of clarity and conciseness, blots were cropped to the bands of interest. Full-length blots are presented in Supplementary Figure S10.

Supplementary Figure S3

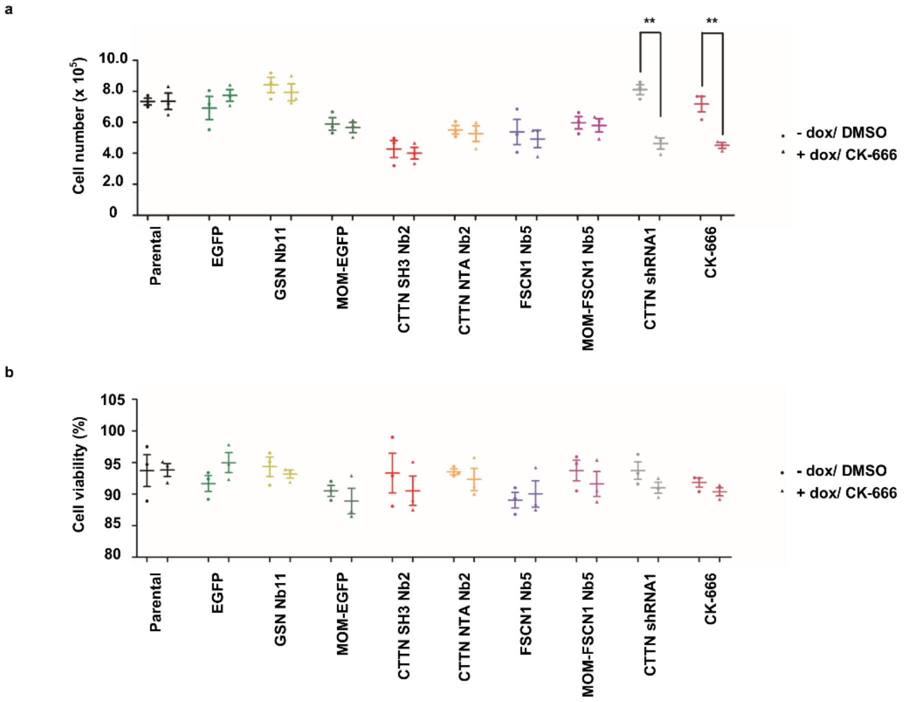

Supplementary Figure S3. CTTN shRNA1 expression and CK-666-treatment reduce cell numbers but do not affect viability. (a) Cells were counted after 48 h in serum-free medium. Dots or triangles represent cell numbers (three independent measurements, n = 3) of non-expressing - dox/ DMSO-treated versus expressing + dox/ CK-666-treated MDA-MB-231, respectively. Graphs represent mean cell number with SEM. $P_{0.05}$-value was determined by a two-sided t test. CTTN shRNA1 expression (p = 0.0019) and CK-666-treatment (p = 0.0075) significantly reduce cell numbers, compared to their non-induced or DMSO-treated equivalents, respectively. ** $P \leq 0.01$. **(b)** Cell viability after 48 h in serum-free medium. Dots or triangles represent % of cells excluding trypan blue staining (three independent measurements, n = 3) of non-expressing - dox/ DMSO-treated versus expressing + dox/ CK-666-treated MDA-MB-231, respectively. Graphs represent mean viability with SEM. $P_{0.05}$-value was determined by a two-sided t test. None of the tested conditions significantly affected cell viability.

Supplementary Figure S4

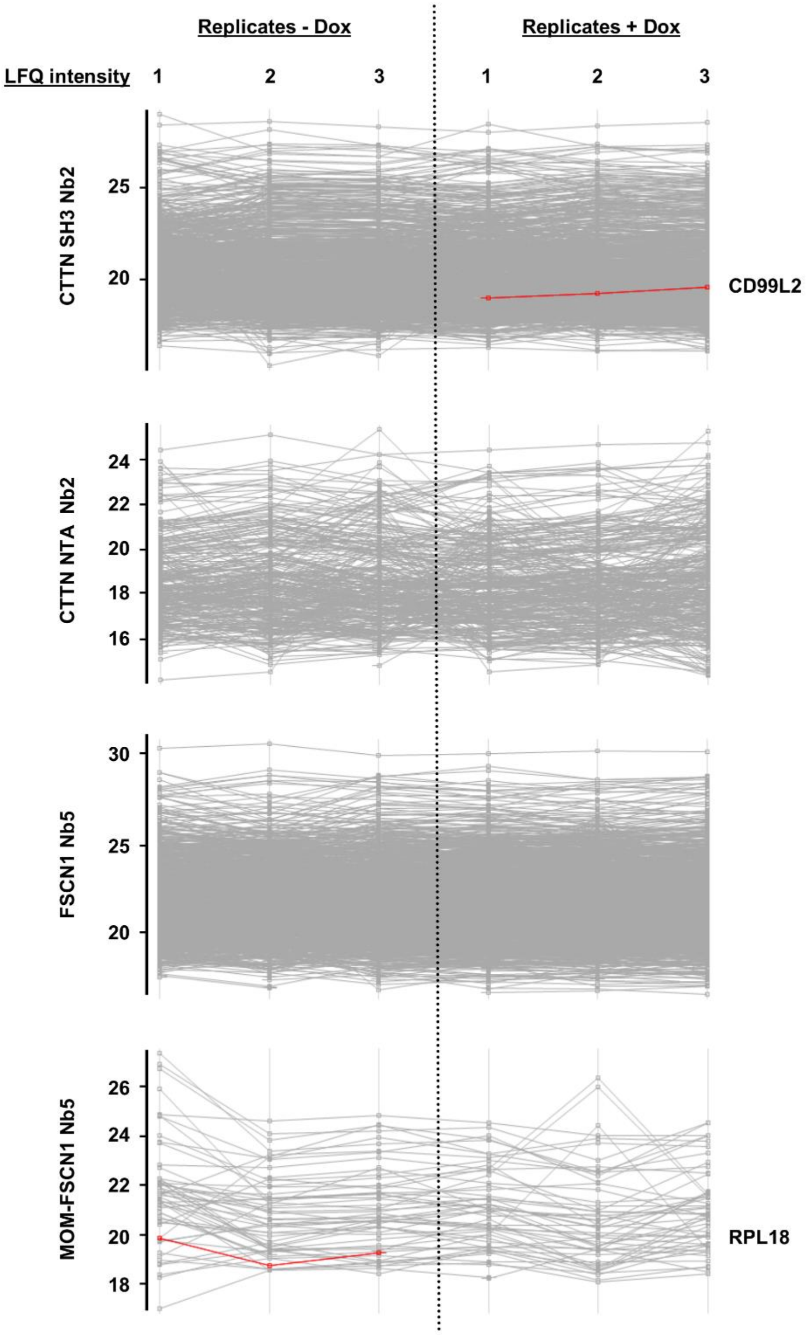

Supplementary Figure S4. CTTN SH3 Nb2 and MOM-FSCN1 Nb5 expression possibly alter protein sorting into MDA-MB-231 EVs. Shotgun MS and following label-free quantification (LFQ) detects CD99 antigen-like protein 2 (CD99L2) in EVs of CTTN SH3 Nb2 expressing cells, whereas the protein was not detected in the corresponding non-expressing cell line. On the other hand, 60S ribosomal protein L18 (RPL18) was no longer detected by MS in EVs isolated from MOM-FSCN1 Nb5-expressing cells, compared to EVs of non-expressing cells. EV composition does not differ between expressing and non-expressing

CTTN NTA Nb2 or FSCN1 Nb5 cells. Graphs represent profile plots of LOG2(x)-converted LFQ intensities of each biological replicate (n = 3). Only proteins detected in three replicates of at least one group (i.e. - dox or + dox) are shown in the graph.

Supplementary Figure S5

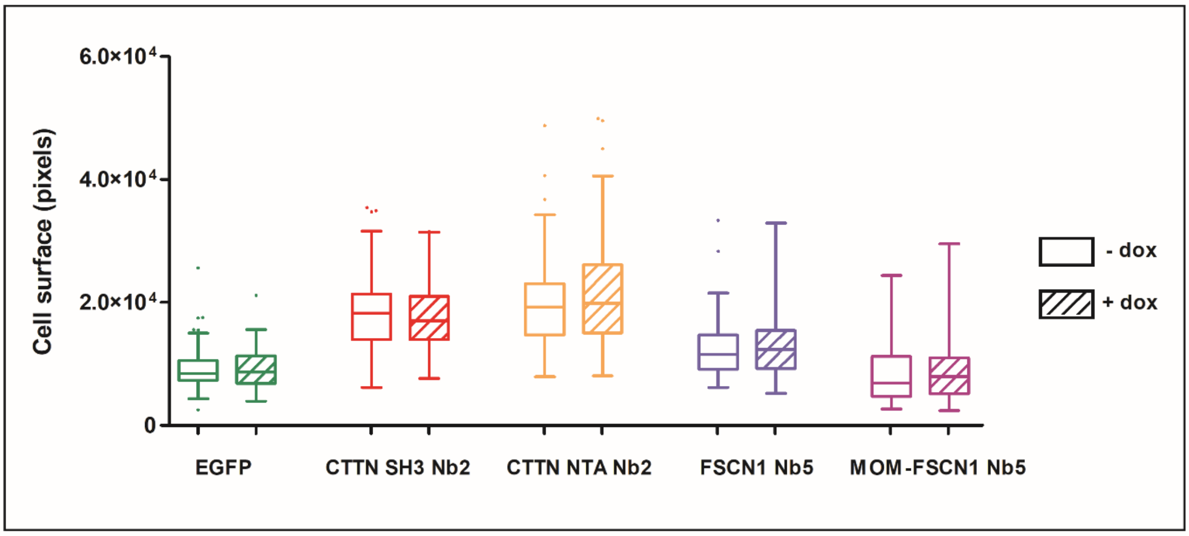

Supplementary Figure S5. Nanobody expression does not significantly alter cell size. Cell cortex was marked out using Acti-Stain 670 phalloidin staining in MDA-MB-231 cells without (- dox) or with (+ dox, 24 h, 500 ng/ ml dox) nanobody expression. Tukey boxplots represent quantification of total cell area. $P_{0.05}$-value was determined by a Mann-Whitney rank sum test and n ≥ 100 cells were quantified for each boxplot. Cell size does not significantly change when nanobody or EGFP are expressed compared to the corresponding non-expressing cell line. P-values: 0.9521 (EGFP), 0.4401 (CTTN SH3 Nb2), 0.3589 (CTTN NTA Nb2), 0.8960 (FSCN1 Nb5), 0.3326 (MOM-FSCN1 Nb5).

Supplementary Figure S6

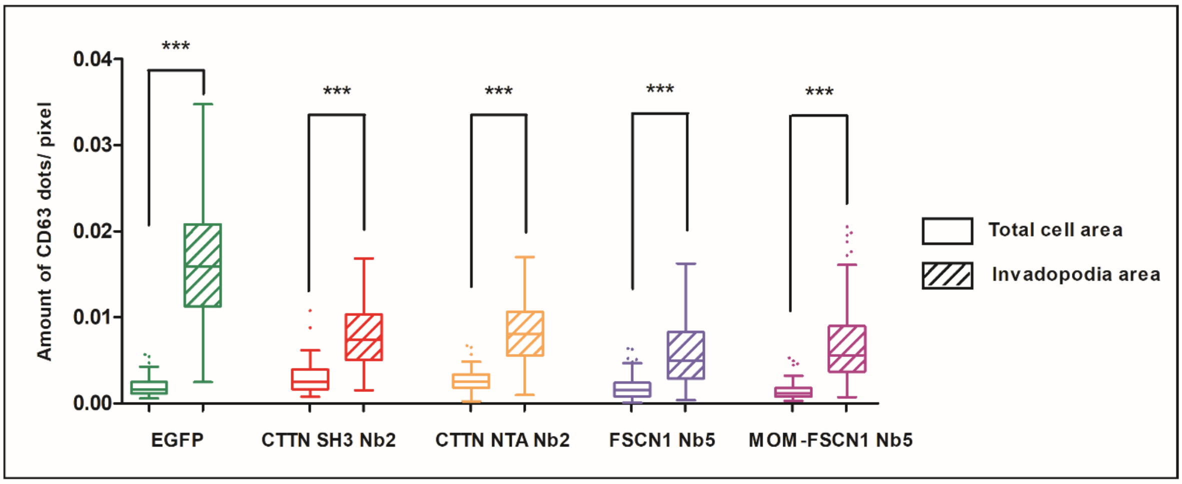

Supplementary Figure S6. CD63+ vesicles localize near invadopodia in nanobody-expressing MDA-MB-231. Quantification of the CD63-dot density at the total cell area versus phalloidin (Acti-Stain 670 phalloidin) perinuclear region. Tukey boxplots represent quantification of the amount of CD63 dots per pixel. $P_{0.05}$-value was determined by a Mann-Whitney rank sum test and n ≥ 100 cells were quantified for each boxplot. CD63-dot density is significantly higher at the perinuclear phalloidin area compared to the total cell area in EGFP and nanobody-expressing cells ($p < 0.0001$). *** $P \leq 0.001$.

Supplementary Figure S7

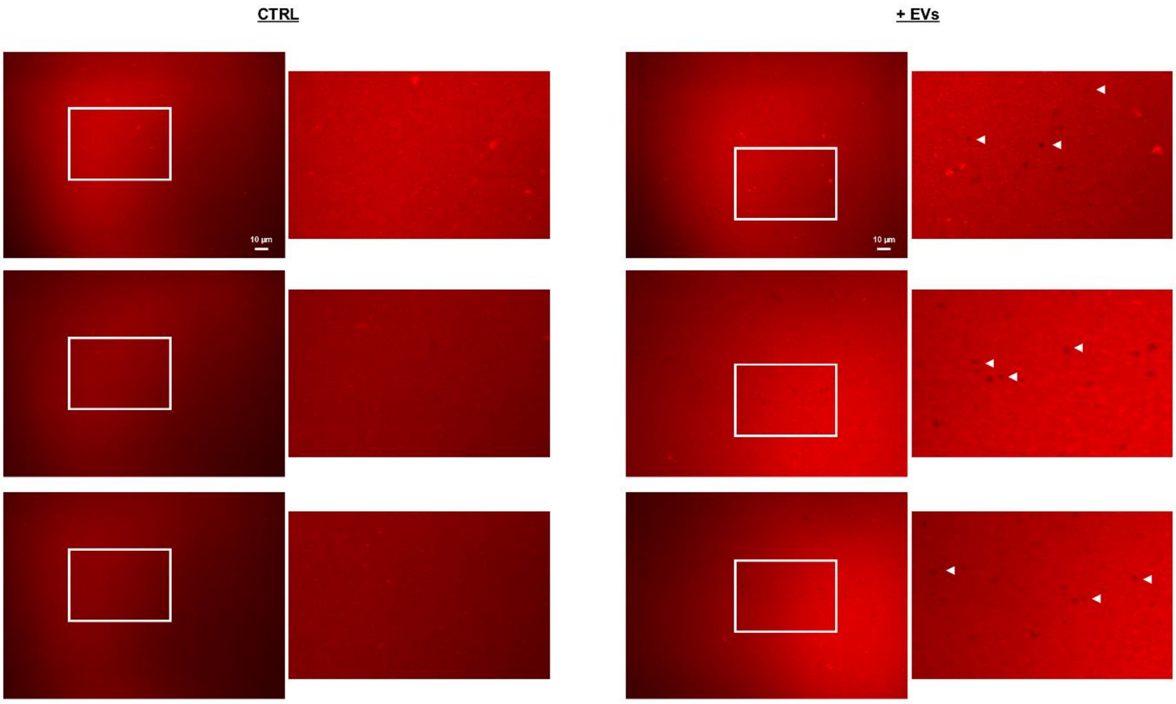

Supplementary Figure S7. Representative confocal images of fluorescent gelatin matrices without (CTRL) or with EV-incubation (+ EVs). Representative images of three independent experiments (n = 3). Typical areas of matrix degradation can be observed post-EV incubation (+ EVs), which are completely absent in the control (CTRL) conditions. Boxed areas are enlarged at the right, arrowheads △ indicate areas of degradation.

Supplementary Figure S8

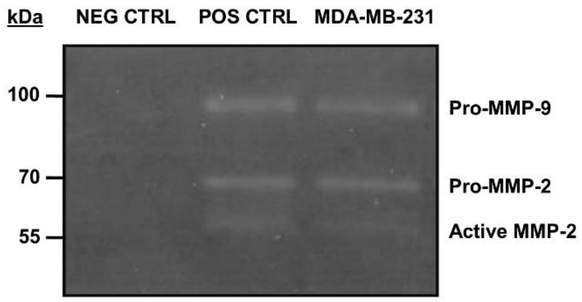

Supplementary Figure S8. MDA-MB-231 secrete MMP-9 and MMP-2. Representative gelatin zymogram on 15 h conditioned HT-1080 (positive control, POS CTRL) or MDA-MB-231 medium. A negative control (NEG CTRL) was included using serum-free DMEM medium. MDA-MB-231 secrete both matrix metalloproteinases MMP-9 and MMP-2.

Supplementary Figure S9

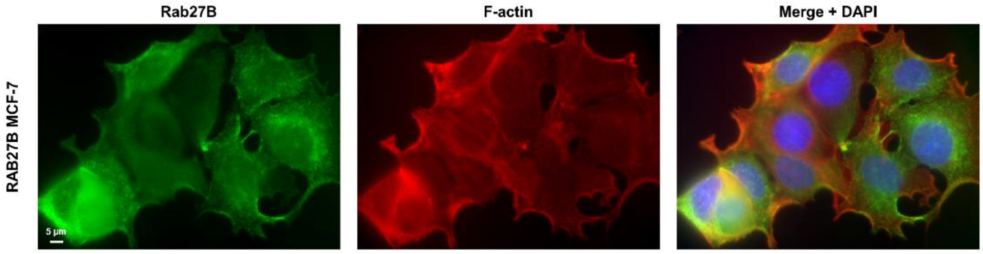

Supplementary Figure S9. RAB27B MCF-7 do not form invadopodia on gelatin matrix. Representative epifluorescence images of RAB27B-overexpressing MCF-7 breast cancer cells seeded onto a gelatin matrix (24 h post-seeding). No invadopodia could be detected (immunofluorescence of F-actin, Alexa Fluor 594 phalloidin staining). Nuclei (blue) were visualized by means of DAPI and RAB27B (green) is intrinsically fluorescent via its EGFP-tag.

Supplementary Figure S10

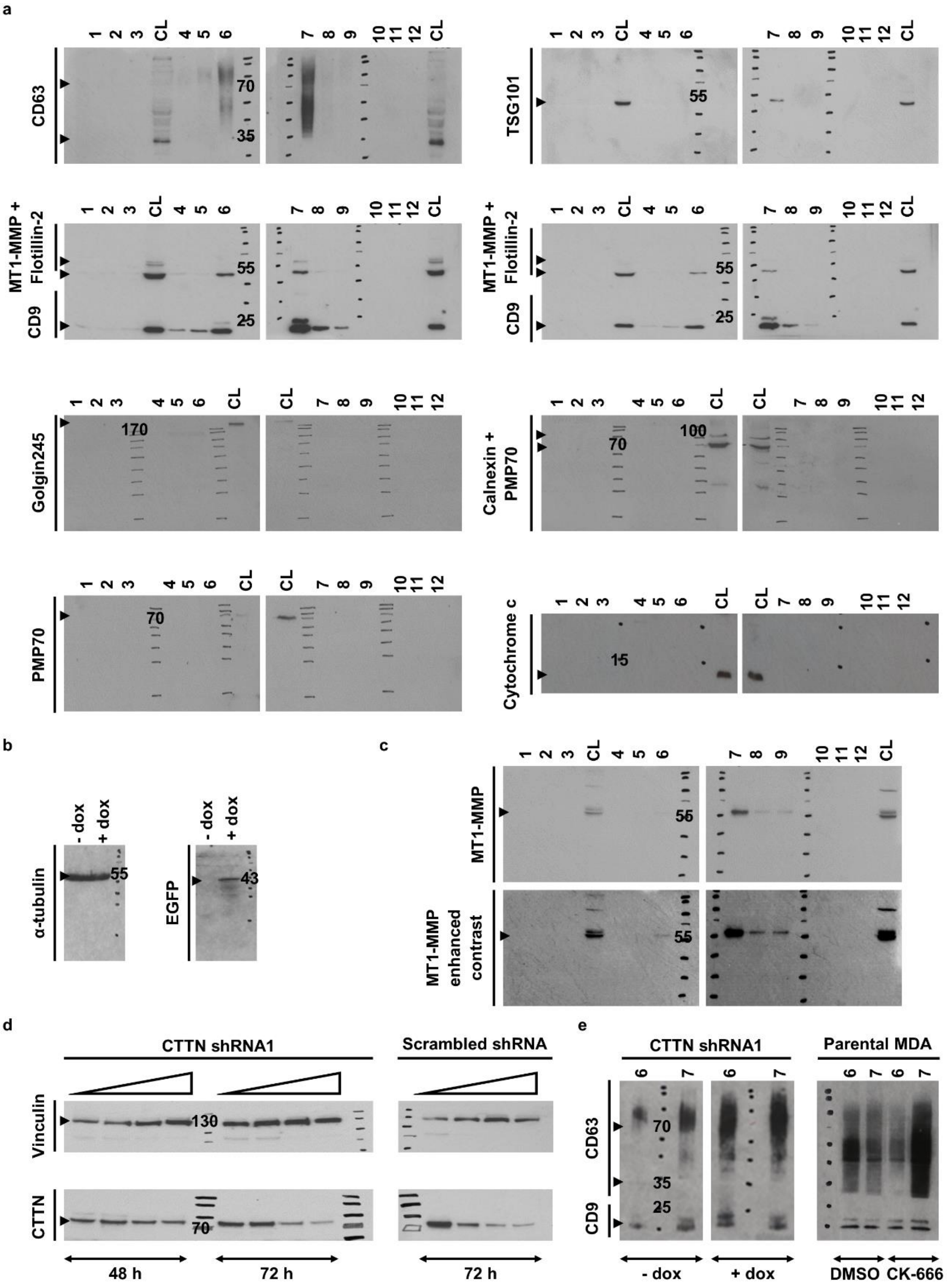

Supplementary Figure S10. Full Western blots of the experiments shown in **(a)** Figure 1b, **(b)** Figure 2a, **(c)** Figure 6d, **(d)** Supplementary Figure S1 and **(e)** Supplementary Figure S2. Uncropped images are labelled as in the main text and arrowheads △ indicate protein bands of interest.

SUPPLEMENTARY METHODS

Generation of actin binding protein (ABP) nanobodies

Nanobodies were obtained in collaboration with the Vlaams Instituut voor Biotechnologie (VIB) Nanobody Service Facility as previously described[1-3]. Briefly, a llama or alpaca was immunized by subcutaneous injection of the antigen on days 0, 7, 14, 21, 28 and 35. On day 39, anticoagulated blood was collected and lymphocytes were isolated. A nanobody library was constructed and screened for the presence of antigen-specific nanobodies. Therefore, total RNA from peripheral blood lymphocytes was used as template for first strand cDNA synthesis with oligodT primers. Using this cDNA, the nanobody encoding sequences were amplified by PCR and cloned into the PstI and NotI sites of the phagemid vector pHEN4. Subsequently, multiple rounds of phage panning were performed on solid-phase coated antigen in order to isolate antigen-specific nanobodies. The enrichment for antigen-specific phages was assessed after each round of panning by comparing the number of phagemid particles eluted from antigen-coated wells with the number eluted from wells coated with blocking solution only. Individual colonies were analysed by ELISA for the presence of antigen-specific nanobodies in their periplasmic extracts. Finally, positive colonies were analysed by nucleotide sequencing. All nanobodies used in this study were previously thoroughly characterized: gelsolin Nb11 (GSN Nb11)[4], cortactin SH3 Nb2 (CTTN SH3 Nb2)[1,2], CTTN NTA Nb2[2], fascin-1 Nb5 (FSCN1 Nb5)[1,3] and MOM-FSCN1 Nb5[1].

Antibodies, reagents and buffer preparations

Rabbit monoclonal anti-CD63 (ab134045, Western blot or WB 1:1000) mouse monoclonal anti-CD63 (ab8219, immunocytochemistry or IC 1:100), rabbit polyclonal anti-PMP70 (ab85550, WB 1:500), mouse monoclonal anti cytochrome c (ab13575, WB 1:1000) and rabbit monoclonal MT1-MMP (ab51074, WB 1:2000) were obtained from Abcam (Cambridge, UK). Rabbit monoclonal anti-CD9 (#13174, WB 1:1000) was purchased from Cell Signaling Technology (Danvers, MA, USA). Mouse monoclonal anti-TSG101 (sc-7964, WB 1:1000), mouse monoclonal anti-flotillin-2 (sc-28320, WB 1:500), rabbit polyclonal anti-golgin-245 (sc-102565, WB 1:1000, IF 1:200), rabbit polyclonal anti-calnexin (sc-11397, WB 1:1000) and rabbit polyclonal anti-TKS5 (sc-30122, IF 1:100) were obtained from Santa Cruz Biotechnology (Santa Cruz, CA, USA). Mouse monoclonal anti-vinculin (V9131, WB 1:1000) and mouse monoclonal anti-α-tubulin (T6199, WB 1:1000) were obtained from Merck (Billerica, Massachusetts, USA). Alexa Fluor 488/ 594 goat anti-mouse or Alexa Fluor 594 goat anti-rabbit were purchased from Thermo Fisher Scientific (Waltham, MA, USA). ECL anti-mouse or rabbit IgG horseradish peroxidase-linked substrates were purchased from GE Healthcare. ARP2/3-inhibitor CK-666 (182515), bovine skin gelatin (G1393), glutaraldehyde

(G6257), QCM Gelatin Invadopodia Assay kit (ECM671) and DAPI (D9542, IC 1:1000) were from Merck. Alexa Fluor 594 phalloidin (A12381, IC 1:100) was purchased from Thermo Fisher Scientific. Acti-Stain 670 phalloidin (PHDN1-A, IC 1:50) was obtained from Cytoskeleton (Denver, CO, USA). Rat tail collagen type I (#354249) was purchased from Corning (Bedford, MA, USA). OptiPrep (60 % w/v solution of iodixanol in water) was purchased from Axis-Shield (Oslo, Norway). OptiPrep gradient solutions were prepared as followed: 1 volume of working solution buffer (0.25 M sucrose, 6 mM EDTA and 60 mM Tris-HCl pH 7.4) was mixed with 5 volumes of OptiPrep, resulting in a 50 % dense working solution. This working solution was further diluted with homogenization medium (0.25 M sucrose, 1 mM EDTA and 10 mM Tris-HCl pH 7.4) to provide the 40 %, 20 %, 10 % and 5 % OptiPrep gradient solutions used in the EV isolation protocol.

cDNA cloning

Nanobodies were equipped with a C-terminal EGFP and/ or N-terminal MOM (Mitochondrial Outer Membrane)-tag and subsequently cloned into the doxycycline (dox)-inducible lentiviral expression pLVX-Tight-Puro vector (Clontech, Mountain View, CA, USA) as described previously[1]. Short hairpin RNA targeting CTTN TRCN0000040273 (shRNA library of the Broad Institute, Genetic Perturbation Platform) in the pLKO.1 backbone vector was kindly provided by Prof. Dr. Geert Berx (Molecular Cellular Oncology Lab, Department for Biomedical Molecular Biology, Ghent University, Ghent, Belgium). In order to generate a dox-inducible construct, the short hairpin sequence was subcloned into pLKO-Tet-On using following primers: 5’ ATCAGTGATAGAGACACCGGTCGGCAAATACGGTATCGACA 3’ (forward) and 5’ TTGTCTCGAGGTCGAGAATTCCAAAAACGGCAAATACGGTA 3’ (reverse) and utilizing the Cold Fusion Cloning Kit from System Biosciences (Palo Alto, CA, USA). The resulting vector is referred to as CTTN shRNA1 throughout the manuscript. Control Scrambled shRNA in pLKO-Tet-On was a kind gift of Prof. Dr. Geert Berx.

Cell culture and transduction

MDA-MB-231, MCF-7, RAB27B MCF-7, HT-1080 and HEK293T cells were grown at 37 °C in a humidified 10 % CO_2 incubator. All cells were cultured in DMEM supplemented with 10 % foetal bovine serum and 10 U/ ml penicillin-streptomycin (all from Gibco, Thermo Fisher Scientific). Cell cultures were frequently tested and found negative for mycoplasma contamination using the HEK-Blue detection kit from Invivogen (San Diego, CA, USA).

RAB27B MCF-7 were a kind gift of Prof. Dr. Olivier De Wever (Laboratory of Experimental Cancer Research, Department of Human Structure and Repair, Ghent University, Ghent, Belgium). MDA-MB-231 cells stably expressing EGFP, MOM-EGFP, gelsolin Nb11-EGFP (GSN Nb11), cortactin SH3 Nb2-EGFP (CTTN SH3 Nb2), cortactin NTA Nb2-EGFP (CTTN

NTA Nb2), fascin-1 Nb5-EGFP (FSCN1 Nb5), MOM-fascin-1 Nb5-EGFP (FSCN1 Nb5), cortactin shRNA1 (CTTN shRNA1) and Scrambled shRNA were created using the Lenti-X Tet-On Advanced Inducible Expression System (Clontech) as previously described[1]. In brief, HEK293T were calcium phosphate-transfected with packaging (psPAX2), envelope (pMD2.G) and nanobody (pLVX-Tight-Puro) or shRNA (pLKO-Tet-On) plasmids. Medium with lentiviral particles was collected 48 h and 72 h post-transfection, filtered (0.45 µm) and concentrated by means of ultracentrifugation. Viral titre was determined by crystal violet staining on transduced and puromycin-selected HEK293T cells. Next, MDA-MB-231 cells were infected at an MOI = 5 with regulator and nanobody virus and subjected to puromycin (nanobody) and G418 (regulator) selection for 3 weeks. In case of shRNA, MDA-MB-231 cells were infected (MOI = 5) with shRNA virus alone and subsequently subjected to puromycin selection for 3 weeks. Except for GSN Nb11, CTTN shRNA1 and Scrambled shRNA MDA-MB-231, all cell lines were fully described beforehand and have known effects on invadopodium formation, lifetime and maturation[1-3].

Validation of stable shRNA MDA-MB-231

Scrambled shRNA and CTTN shRNA1 expressing MDA-MB-231 were incubated with different concentrations of dox (0, 10, 100 or 500 ng/ ml) during 48 h or 72 h. Next, cells were lysed in ice-cold lysis buffer (0.5 % NP-40, 1 mM PMSF, 1 mM protease inhibitor cocktail, 150 mM NaCl and 20 mM Tris HCl pH 7.5 in MQ), sonicated and pelleted. Protein concentrations were determined using the Bradford assay (Bio-Rad Laboratories, Hercules, CA, USA). 60 µg crude lysate each condition was mixed with reducing Laemmli SDS sample buffer (65 mM Tris- HCl pH 6.8, 20 % glycerol, 5 % SDS, 0.2 % bromophenol blue, 5 % β-mercaptoethanol in Milli-Q) followed by SDS-PAGE and Western blot analysis.

Assessment of cell numbers and viability

4×10^5 (parental MDA-MB-231, EGFP, MOM-EGFP, MOM-FSCN1 Nb5) or 2.5×10^5 (GSN Nb11, CTTN SH3 Nb2, CTTN NTA Nb2, FSCN1 Nb5) cells were seeded in six-well plates. On day 2, medium was changed and cells were further grown on serum-free medium (n = 3 each cell line) or serum-free medium supplemented with 500 ng/ ml dox, 100 µM CK-666 or DMSO (n = 3 each cell line). Medium was again changed 24 h later (with or without dox/ CK-666/ DMSO). On day 4, cells were trypsinized. Viability (trypan blue staining) and cell numbers were assessed using a Luna-II automated cell counter (Logos Biosystems, Gyeonggi-do, South Korea). In case of CTTN shRNA1 cell line, 1.5×10^5 cells were seeded and induced 72 h before they were grown on serum-free medium (again with or without dox). Data were analysed using a two-sided t test ($n = 3$, $\alpha = 0.05$, Shapiro-Wilk normality test passed) using

GraphPad software, for each cell line comparing its induced + dox/ CK-666 to its non-induced - dox/ DMSO control (identical colours on the graph), respectively.

EV characterization

EV density

EV density ρ was determined according to the protocol mentioned in[5]. Briefly, a standard curve was made of absorbance values at 340 nm of 1:1 aqueous dilution of OptiPrep gradient solutions. This standard curve was used to estimate the w/v % of each fraction collected from a control gradient overlaid with 1 ml of PBS (without Ca^{2+} and Mg^{2+}). These estimated values could then be used to calculate the density of the fractions, according to the manufacturer's instructions published in OptiPrep Application Sheet V01.

EV protein analysis by Western blot

20 µl of each fraction following EV isolation was mixed with reducing Laemmli SDS sample buffer (65 mM Tris- HCl pH 6.8, 20 % glycerol, 5 % SDS, 0.2 % bromophenol blue, 5 % β-mercaptoethanol in Milli-Q) and examined for presence of EV-markers CD63, TSG101, flotillin-2 and CD9. For the detection of potentially contaminating proteins golgin-245, calnexin, PMP70 and cytochrome c, fractions were additionally sonicated in an ultrasonic bath for 1 h.

EV protein analysis by shotgun mass spectrometry (MS)

EV+ fractions were analysed for protein content using shotgun MS and in collaboration with the Proteomics Expertise Center (VIB Proteomics Core, VIB-UGent Center for Medical Biotechnology, Department of Biomolecular Medicine, Ghent University, Ghent, Belgium). Briefly, equal amounts of EV+ fractions were mixed to a total volume of 30 µl. Proteins were denatured using a urea lysis buffer (8 M urea in 20 mM HEPES, pH 8), followed by reduction (15 mM DTT) and alkylation (30 mM iodoacetamide) of cysteine bridges and protein digestion with endoproteinases lys-C (Wako Chemicals, Richmond, VA, USA) and trypsin (Promega, Madison, WI, USA) (each time 1:100 weight proteinase: weight sample). The resulting peptide mixture was desalted on C18 OMIX tips (Agilent, Santa Clara, CA, USA) prior to LC-MS/MS analysis. Samples prepared from parental MDA-MB-231 were analysed by means of a Q-Exactive Quadrupole-Orbitrap (Thermo Fisher Scientific). Samples prepared from CTTN SH3 Nb2, CTTN NTA Nb2, FSCN1 Nb5 and MOM-FSCN1 Nb5 were analysed using a LTQ Orbitrap Elite (Thermo Fisher Scientific). All MS data were searched using MAXQUANT (version 1.6.1.0) against the human SwissProt database (September 2017). Identifications against the REVERSE database and potential contaminants were removed using PERSEUS software (version 1.6.0.7), followed by LOG-transformation (base 2). For the comparison of

EV content between nanobody-expressing (+ dox) versus non-expressing (- dox) cells, three biological replicates (corresponding to the replicates used for nanoparticle tracking analysis) of each group (- dox and + dox) were label-free quantified. Only proteins detected in three replicates of at least one group were selected for further quantification.

Gelatin zymography

1 x 10^6 HT-1080 and MDA-MB-231 cells were seeded in a 1 mg/ ml collagen matrix that was allowed to polymerize for 1 h at 37 °C. After 15 h incubation in serum-free medium, 2 µg of conditioned medium proteins were analysed by means of gelatin zymography, as was described before[1].

SUPPLEMENTARY REFERENCES

1. Van Audenhove, I. *et al.* Stratifying fascin and cortactin function in invadopodium formation using inhibitory nanobodies and targeted subcellular delocalization. *FASEB journal : official publication of the Federation of American Societies for Experimental Biology* **28**, 1805-1818, doi:10.1096/fj.13-242537 (2014).
2. Bertier, L. *et al.* Inhibitory cortactin nanobodies delineate the role of NTA- and SH3-domain-specific functions during invadopodium formation and cancer cell invasion. *FASEB journal : official publication of the Federation of American Societies for Experimental Biology*, doi:10.1096/fj.201600810RR (2017).
3. Van Audenhove, I. *et al.* Fascin Rigidity and L-plastin Flexibility Cooperate in Cancer Cell Invadopodia and Filopodia. *The Journal of biological chemistry*, doi:10.1074/jbc.M115.706937 (2016).
4. Van den Abbeele, A. *et al.* A llama-derived gelsolin single-domain antibody blocks gelsolin-G-actin interaction. *Cellular and molecular life sciences : CMLS* **67**, 1519-1535, doi:10.1007/s00018-010-0266-1 (2010).
5. Van Deun, J. *et al.* The impact of disparate isolation methods for extracellular vesicles on downstream RNA profiling. *J Extracell Vesicles* **3**, DOI 10.3402/jev.v3403.24858, doi:10.3402/jev.v3.24858 (2014).
